# Dopaminergic Receptors on CD4+ T Naive and Memory Lymphocytes Correlate with Motor Impairment in Patients with Parkinson’s Disease

**DOI:** 10.1038/srep33738

**Published:** 2016-09-22

**Authors:** Natasa Kustrimovic, Emanuela Rasini, Massimiliano Legnaro, Raffaella Bombelli, Iva Aleksic, Fabio Blandini, Cristoforo Comi, Marco Mauri, Brigida Minafra, Giulio Riboldazzi, Vanesa Sanchez-Guajardo, Franca Marino, Marco Cosentino

**Affiliations:** 1 Center of Research in Medical Pharmacology, University of Insubria, Varese, Italy; 2 Center for Research in Neurodegenerative Diseases, “C. Mondino”, National Neurological Institute, Pavia, Italy; 3 Movement Disorders Centre, Neurology Unit, Department of Translational Medicine, University of Piemonte Orientale, Novara, Italy; 4 Department of Biotechnology and Life Sciences, University of Insubria, Varese, Italy; 5 Neuroimmunology of Degenerative Diseases group and AUidias pilot-center NEURODIN, department of Biomedicine, HEALTH, Aarhus University, Aarhus, Denmark.

## Abstract

Parkinson’s disease (PD) is characterized by loss of dopaminergic neurons in substantia nigra pars compacta, α-synuclein (α-syn)-rich intraneuronal inclusions (Lewy bodies), and microglial activation. Emerging evidence suggests that CD4+ T lymphocytes contribute to neuroinflammation in PD. Since the mainstay of PD treatment is dopaminergic substitution therapy and dopamine is an established transmitter connecting nervous and immune systems, we examined CD4+ T naive and memory lymphocytes in PD patients and in healthy subjects (HS), with specific regard to dopaminergic receptor (DR) expression. In addition, the *in vitro* effects of α-syn were assessed on CD4+ T naive and memory cells. Results showed extensive association between DR expression in T lymphocytes and motor dysfunction, as assessed by UPDRS Part III score. In total and CD4+ T naive cells expression of D_1_-like DR decrease, while in T memory cells D_2_-like DR increase with increasing score. *In vitro*, α-syn increased CD4+ T memory cells, possibly to a different extent in PD patients and in HS, and affected DR expression with cell subset-specific patterns. The present results support the involvement of peripheral adaptive immunity in PD, and may contribute to develop novel immunotherapies for PD, as well as to better use of current dopaminergic antiparkinson drugs.

Parkinson’s disease (PD) is the second most common neurodegenerative disorder, affecting an estimated 7 to 10 million people worldwide and resulting in both motor and cognitive disturbances^1^,^2^,^3^. The main neuropathological features of PD are the progressive loss of dopaminergic neurons in the substantia nigra pars compacta, the appearance of intraneuronal inclusions called Lewy bodies, and the occurrence of microglial activation. Microglial cells in particular are key players in neuroinflammation and neurodegeneration, and peripheral adaptive immunity has been recently proposed as a major determinant in the regulation of microglial function during neurodegenerative disease^4^,^5^,^6^,^7^.

Over the last 15 years, several studies described the occurrence of peculiar modifications of peripheral immunity in PD, such as decreased CD4+ /CD8+ T-cell ratios, fewer CD4+ CD25+ T cells and increased ratios of interferon (IFN)-γ-producing to interleukin (IL)-4-producing T cells^8^, as well as decreased CD4+ T lymphocytes and CD19+ B cells^9^,^10^. Both CD8+ and CD4+ T cells (but not B cells) were identified in the brain in both postmortem human PD specimens and in the 1-methyl-4-phenyl-1,2,3,6-tetrahydropyridine (MPTP) mouse model of PD, and evidence obtained in the animal model pointed to CD4+ T cells as main determinants of T cell-mediated dopaminergic cell death^11^. Remarkably, Saunders *et al*.^12^ recently reported that PD patients have increased effector/memory CD4+ T cells and decreased CD31+ and α4β7+ CD4+ T cells, which are associated with progressive motor dysfunction, suggesting a direct relationship between chronic immune stimulation and PD neuropathology and disease severity, as well as strengthening the idea that in PD the lead actors among adaptive immune system cells are CD4+ T lymphocytes.

No therapies are currently available for the neurodegenerative processes underlying PD, and symptomatic treatments rely on the dopamine (DA) precursor l-DOPA as well on dopaminergic agonists and on other indirect dopaminergic agents. Dopaminergic drugs relieve to some extent the loss of brain dopaminergic neurons occurring in PD, although, as disease progresses, both nonmotor and motor symptoms emerge that are resistant to dopaminergic medications^13^. Interestingly DA, besides its role as brain neurotransmitter, is also an established transmitter connecting the nervous and the immune system, as well as immune cells and peripheral tissues^14^,^15^,^16^. DA affects both innate and adaptive immune system cells, and immune cells produce DA, which may act as autocrine/paracrine mediator on immune cells themselves and on neighboring cells^17^,^18^,^19^,^20^,^21^,^22^. Among T lymphocytes, CD4+ T cells may represent a major target for DA. DA subserves an (auto)inhibitory loop in human CD4+ CD25^high^ regulatory T lymphocytes, a specialized T cell subset playing a key role in the control of immune homeostasis^23^, and dendritic cells-derived DA affects the differentiation of naive CD4+ T cells^24^. The effects of DA are exerted through 5 different dopaminergic receptors (DR) grouped into the D_1_-like (D_1_ and D_5_) and the D_2_-like (D_2_, D_3_ and D_4_)^25^,^26^. Immune cells express all DR and in particular CD4+ T cells express both D_1_-like and D_2_-like DR^23^,^27^, with CD4+ naive T cells expressing more D_1_-like than D_2_-like DR, which on the contrary are increased in memory T cells^28^. Despite extensive evidence supporting the involvement of CD4+ T cells (and in particular of memory T cells) in PD pathogenesis and progression^8^,^10^,^11^,^12^, no information exists on DR expression on CD4+ T cells in PD patients. Nonetheless, a recent investigation in the MPTP mouse model of PD suggested that DR D_3_ expressed on CD4+ T cells are critical for T cell-dependent microglial activation, which finally results in neurodegeneration^29^.

The present study, examined CD4+ T cells, as well as naive and memory CD4+ T cell subsets, in PD patients and in healthy subjects, with specific regard to DR expression. Correlations of CD4+ T cell and cell subsets, as well as their respective expression of DR, were investigated with demographic and clinical features of the subjects. Patients on dopaminergic agents were compared with recently diagnosed patients who never received dopaminergic medications. In addition, in preliminary experiments the *in vitro* effects of α-synuclein (α-syn) were assessed on CD4+ T naive and memory cells. α-syn is a protein expressed in brain and in peripheral tissues. It is the main component of Lewy bodies and it may contribute to the pathogenesis of PD through different concurrent mechanisms, including direct activation of microglial cells as well as possibly by acting as an antigen itself, triggering the adaptive immune response in the periphery^30^,^31^,^32^. For these reasons, the effects of α-syn on CD4+ T naive and memory cells were also compared with those of a common recall antigen like tetanus toxoid (TTd).

## Results

### Subjects

We enrolled 53 PD patients and 28 HS (Table 1). Patients comprised 16 subjects who had been never treated with antiparkinson drugs before enrollment, and were therefore drug naive (PD-dn). In comparison to patients on antiparkinson drugs (PD-dt), PD-dn were younger and had on average lower H&Y stage, as well as UPDRS Part III score (Table 2). Plasma dopamine was 3.2 ± 5.7 nM in HS, 2.7 ± 3.3 nM in PD-dn patients (P = 0.779 vs. HS), and 8.0 ± 9.8 nM in PD-dt patients (P = 0.003 vs. HS).

Complete blood counts of PD patients and HS were all within normal limits (Table 3), however PD patients had less total lymphocytes, both in terms of absolute number (on average, about 17% less) and as percentage of white blood cells (−3.5%).

Complete blood count did not differ between PD-dn and PD-dt patients (Table 4), with the only exception of percentage of basophils, which were slightly lower in PD-dt.

### T lymphocytes and CD4+ T naive and memory subsets

Reduction of lymphocytes in PD patients was accounted for essentially by T cells (Table 3). In particular, CD4+ T cells were about 21% less in PD patients in comparison to HS, even if the CD4+ /CD8+ ratio did not change in patients, likely due the overall reduction of T lymphocytes. T lymphocyte subsets did not differ in PD-dn and PD-dt patients in terms of absolute counts, even if in PD-dn patients CD3+ T cells as percentage of total lymphocytes were less (−5.4%) and CD4+ T cells as percentage of CD3+ T cells were more (+6.4%) (Table 4).

**Figure 1:**
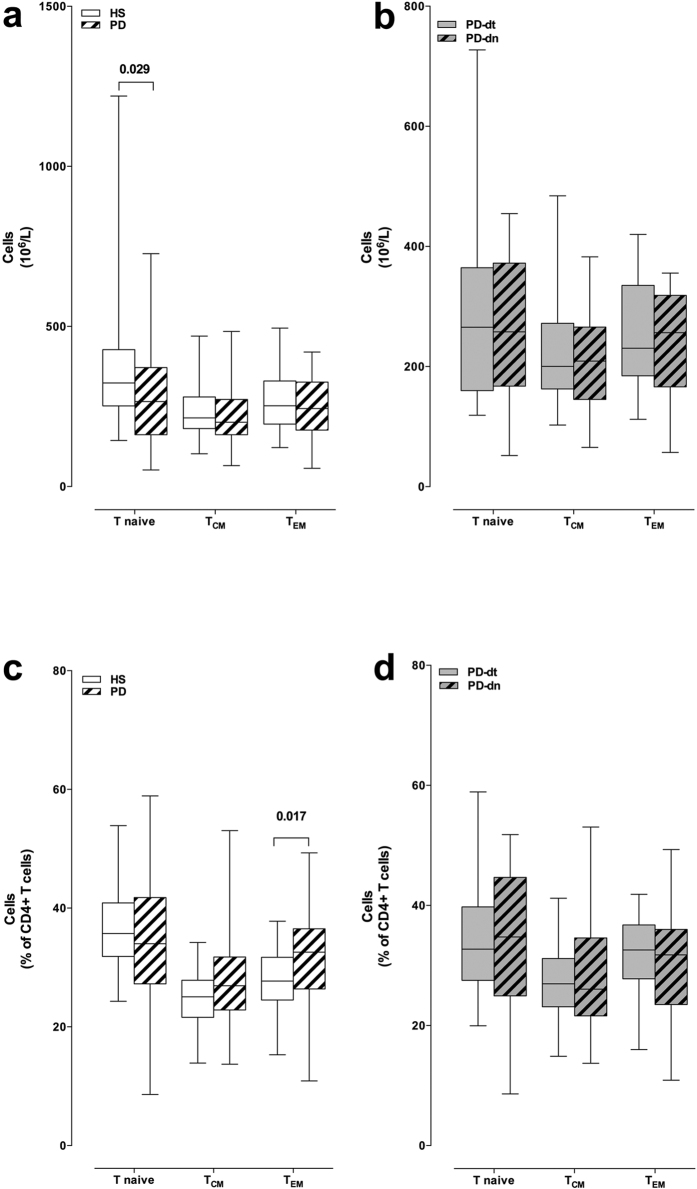
CD4+ T naive and memory cells in HS and PD patients. Cells are shown as absolute numbers (panels **a,b**) and as percentage of total CD4+ T cells (**c,d**). Data are shown as medians with 25°–75° percentiles (boxes) and min-max values (whiskers). Comparisons are shown between HS and PD patients as a whole (**a,c**) and between drug naive (PD-dn) and drug treated (PD-dt) patients (**b,d**). Differences were analyzed by means of two-tailed Student’s *t* test or by Mann-Whitney test, as appropriate. P values less than 0.05 are indicated in the graphs.

To identify CD4+ T cell subsets, the gating strategy included the use of a biparametric dot plot CD45RA vs. CCR7, that allowed the identification of T naive (CD3+ CD4+ CD45RA+ CCR7+ ), T_CM_ (CD3+ CD4+ CD45RA-CCR7+ ), and T_EM_ (CD3+ CD4+ CD45RA-CCR7-)^28^. Among CD4+ T cells, PD patients showed decreased number of T naive cells (Fig. 1a). CD4+ T_CM_ and T_EM_ absolute numbers were not different in PD patients and in HS, however T_EM_ as percentage of total CD4+ T cells were increased in PD patients (+3.3% on average) (Fig. 1c). PD-dn and PD-dt patients did not differ in either absolute number or percentage of T naive, T_CM_ and T_EM_ (Fig. 1b,d).

### DR expression on CD4+ T cells

**Figure 2:**
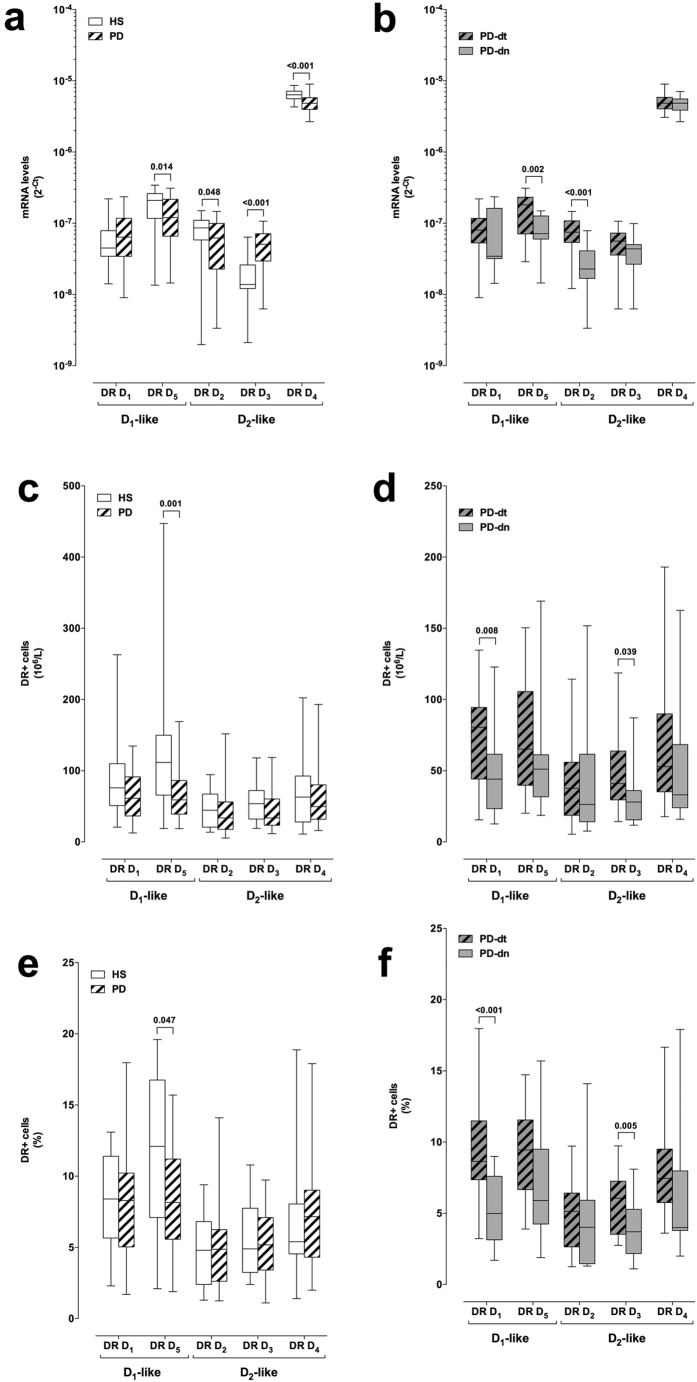
DR expression on CD4+ T cells from HS and from PD patients. DR expression is shown as mRNA levels (panels **a,b**) and as protein expression on the membranes of CD4+ T cells, expressed as absolute numbers of DR+ cells (**c,d**) and as percentage of total CD4+ T cells (**e,f**). Comparisons are shown between HS and PD patients as a whole (**a,c,e**) and between drug naive (PD-dn) and drug treated (PD-dt) patients (**b,d,f**). Data are shown as medians with 25°–75° percentiles (boxes) and min-max values (whiskers). Differences were analyzed by means of two-tailed Student’s *t* test or by Mann-Whitney test, as appropriate. P values less than 0.05 are indicated in the graphs.

DR expression was assessed at both mRNA level (in total CD4+ T cells) and membrane protein level (in total CD4+ T cells and in T naive and memory subsets). In comparison to cells from HS, CD4+ T cells from PD patients had lower mRNA levels of the D_1_-like DR D_5_ and of the D_2_-like DR D_3_ and D_4_, and higher mRNA levels of the D_2_-like DR D_3_, while D_1_-like DR D_1_ mRNA levels were not different between cells from PD patients and HS (Fig. 2a). Interestingly, both the D_1_-like DR D_5_ and the D_2_-like DR D_2_ mRNA levels were lower in cells from PD-dn patients compared to cells from PD-dt patients (Fig. 2b).

Flow cytometric analysis of DR expression on CD4+ T cells revealed that in PD patients DR D_5_+ CD4+ T cells were decreased in terms of both absolute number and percentage of total CD4+ T cells (Fig. 2c,e), and that PD-dn patients had lower number and percentage of DR D_1_+ and D_3_+ CD4+ T cells in comparison to PD-dt subjects (Fig. 2d,f).

### DR expression on CD4+ T naive and memory cells

**Figure 3:**
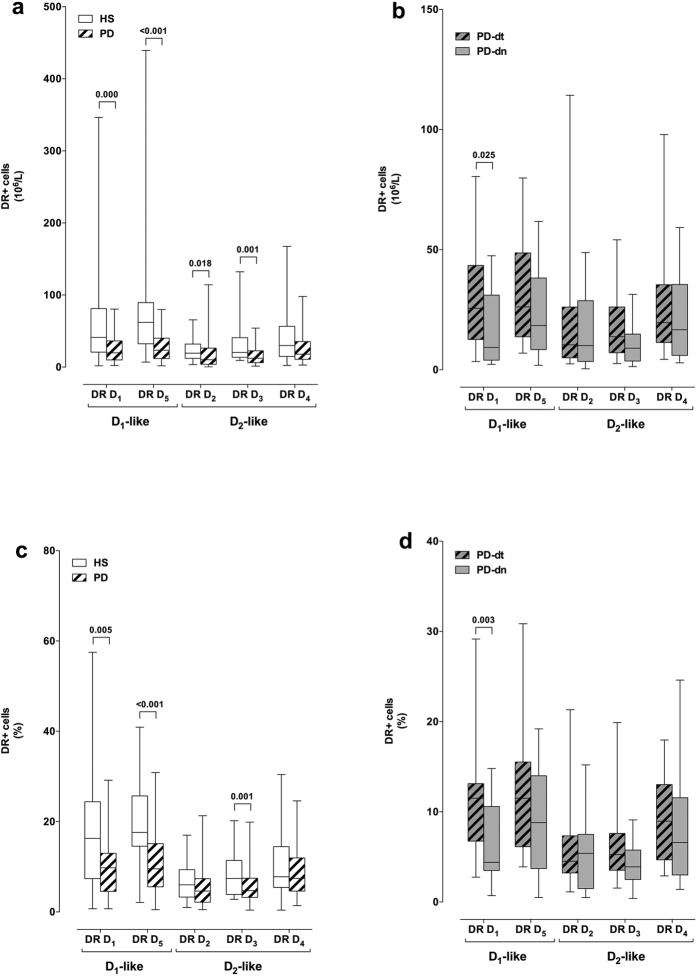
DR expression on CD4+ T naive cells from HS and from PD patients. DR+ cells are shown as absolute numbers (panels **a,b**) and as percentage of total CD4+ cells (**c,d**). Data are shown as medians with 25°–75° percentiles (boxes) and min-max values (whiskers). Comparisons are shown between HS and PD patients as a whole (**a,c**) and between drug naive (PD-dn) and drug treated (PD-dt) patients (**b,d**). Differences were analyzed by means of two-tailed Student’s *t* test or by Mann-Whitney test, as appropriate. P values less than 0.05 are indicated in the graphs.

In comparison to HS, PD patients had less D_1_-like DR D_1_+ and D_5_+, as well as less D_2_-like DR D_2_+ and D_3_+ T naive cells, both in terms of absolute numbers and, for DR D_3_+ cells, also of percentage of total CD4+ T cells (Fig. 3a,c). PD-dn patients had less DR D_1_+ T naive cells in comparison to PD-dt patients, in terms of both absolute numbers and percentage of total CD4+ T cells (Fig. 3b,d).

No difference was found in DR expression in T_CM_ and T_EM_ between PD patients and HS, except for DR D_3_+ T_CM_ which were higher in PD patients in terms of percentage of total CD4+ T cells, and for DR D_4_+ T_EM_ which were higher in PD patients in terms of both absolute numbers and percentage of total CD4+ T cells (see Supplementary Fig. S1 and S2). DR expression on T_CM_ or T_EM_ did not differ between PD-dn and PD-dt patients (see Supplementary Fig. S1 and S2).

### Correlations between CD4+ T cells and demographic and clinical features of HS and PD patients

Age exerts major effects on lymphocyte function^33^, and in particular T naive cells may be reduced in elderly subjects^34^. In agreement with these findings, in HS both the absolute number as well as the percentage of CD4+ T naive cells negatively correlated with age (−0.395 (−0.026 to −0.670), P = 0.037, and −0.472 (−0.120 to −0.718), P = 0.011). No correlation on the contrary was found between age and the immune profile in PD patients, either as a whole or in PD-dn and PD-dt patients.

The relationship between disease severity and CD4+ T cells was assessed by dividing PD patients into 3 groups according either to the UPDRS Part III score or the H&Y stage (Table 1), thereafter comparing each group with HS and analyzing the linear trend throughout the groups by means of ANOVA. No relationship was found between CD4+ T naive or memory cells and the UPDRS Part III score or the H&Y stage, with the only exception of a positive linear trend in the percentage of T_CM_ cells and UPDRS Part III (see Supplementary Fig. S3).

In PD-dt patients, no relationship was found between either CD4+ T cells as a whole or CD4+ T naive or memory cells and disease duration or LED (data not shown).

### Correlations between DR expression on CD4+ T cells and demographic and clinical features of HS and PD patients

**Figure 4:**
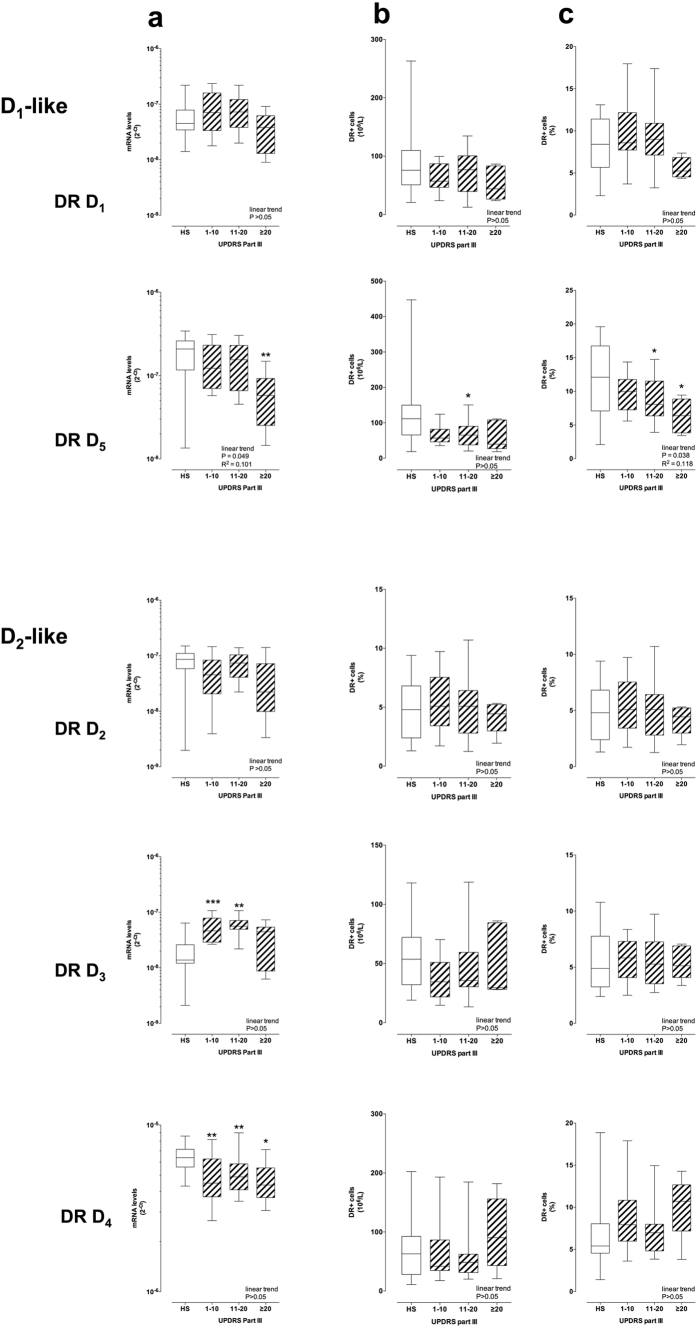
DR expression on CD4+ T cells and UPDRS-III score. DR expression is shown as mRNA levels (panel **a**) and as protein expression on the membranes of CD4+ T cells, expressed as absolute numbers of DR+ cells (**b**) and as percentage of total CD4+ T cells (**c**) Data are medians with 25°–75° percentiles (boxes) and min-max values (whiskers). Differences in DR expression between HS and PD patients were analyzed by parametric ANOVA or Kruskal-Wallis nonparametric ANOVA, as appropriate, with either Holm-Sidak’s or Dunn’s adjustments for multiple comparisons, where * = P < 0.05 and ** = P < 0.01. Trend analysis in PD patients was performed by ANOVA post test for linear trend.

DR mRNA levels in CD4+ T cells showed extensive correlations with the UPDRS Part III score (Fig. 4a). The D_1_-like DR D_5_ mRNA levels decreased with increasing UPDRS Part III scores. In comparison to CD4+ T cells from HS, in cells from PD patients DR D_5_ mRNA levels were lower in the > 20 UPDRS Part III score group. The D_1_-like DR D_1_ and the D_2_-like DR D_2_, D_3_ and D_4_ mRNA levels did not show linear trends over the UPDRS Part III score, however DR D_3_ mRNA levels were higher than those in HS in the 1–10 and 11–20 UPDRS Part III score groups, while DR D_4_ mRNA levels were lower in all the UPDRS Part III score groups (Fig. 4a). Some correlations were also observed between DR mRNA levels and H&Y stage, as DR D_3_ mRNA levels were higher than those in HS in the H&Y 1 and 2 stage groups, while DR D_4_ was lower in H&Y stage 2 (see Supplementary Fig. S4).

Similar correlations with the UPDRS Part III score were observed for DR expression on CD4+ T cell membranes, in the case of DR D_5_ (Fig. 4b,c). Correlations with the H&Y stage included DR D_5_ expression lower than that in HS in the H&Y 2 and 3 stage groups, and DR D_3_ expression lower than that in HS in the H&Y 3 stage groups (see Supplementary Fig. S5).

**Figure 5:**
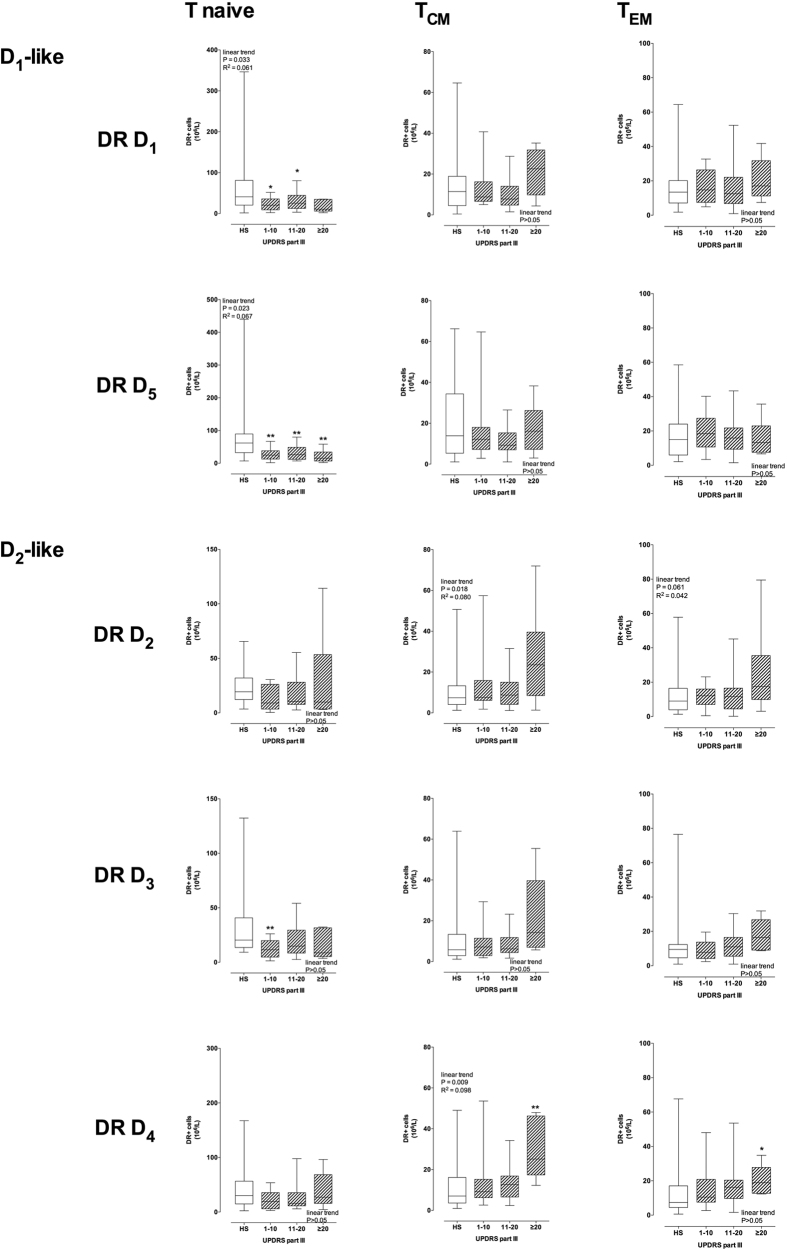
DR expression in CD4+ T naïve, T_CM_ and T_EM_ and UPDRS-III score. DR expression is shown as protein expression on the membranes of CD4+ T naïve (left), T_CM_ (center) and T_EM_ (right) cells, expressed as absolute numbers of DR+ cells. Data are medians with 25°–75° percentiles (boxes) and min-max values (whiskers). Differences between DR levels in HS and in PD patients were analyzed by parametric ANOVA or Kruskal-Wallis nonparametric ANOVA, with either Holm-Sidak’s or Dunn’s adjustments for multiple comparisons, where * = P < 0.05 and ** = P < 0.01. Trend analysis in PD patients was performed by ANOVA post test for linear trend.

D_1_-like DR D_1_ and D_5_ expression on cell membranes negatively correlated with the UPDRS Part III score in CD4+ T naive cells, while D_2_-like DR D_2_, D_3_ and D_4_ didn’t show any major change (Fig. 5). On the contrary, D_2_-like DR D_2_ and D_4_ increased with the UPDRS Part III score in both CD4+ T_CM_ and T_EM_ cells, while D1-like DR did not change either in T_CM_ or T_EM_ (Fig. 5 and Supplementary Fig. S6). Only minor correlations were observed between DR expression and H&Y stage: D_1_-like DR D_1_ and D_5_ and D_2_-like DR D_3_ were reduced in T naive from PD patients in comparison to cells from HS, and D_2_-like DR D_2_ were increased in T_CM_ (see Supplementary Fig. S7-S9).

No correlations were observed between DR mRNA levels and protein expression on CD4+ T cells or DR protein expression in CD4+ T naive and memory cells and age of HS or PD patients, or LED in PD-dt patients. However, in comparison to PD-dt patients treated with l-DOPA and dopamine agonists, those treated with l-DOPA alone had lower mRNA levels of DR D_1_ (6.0 ± 4.1 × 10^−8^ vs. 10.8 ± 5.4 × 10^−8^, P = 0.035), D_5_ (9.7 ± 6.9 × 10^−8^ vs. 20.6 ± 7.5 × 10^−8^, P = 0.003), and D_2_ (6.0 ± 4.4 × 10^−8^ vs. 9.4 ± 3.3 × 10^−8^, P = 0.050), as well as less percentage of CD4+ T cells which were DR D_1_+ (7.2 ± 2.5% vs. 10.3 ± 3.5%, P = 0.025) or DR D_3_+ (4.5 ± 1.5% vs. 6.4 ± 1.9%, P = 0.017), and of CD4+ T naive cells which were DR D_3_+ (3.5 ± 1.2% vs. 7.9 ± 4.6 &, P = 0.009). Patients treated with l-DOPA alone had also higher UPDRS Part III score (19.0 ± 4.3 vs. 14.8 ± 5.0, P = 0.028) but similar H&Y stage (1.8 ± 0.7 vs. 1.8 ± 0.5, P = 0.933).

### *In vitro* responses of CD4+ T naive and memory cells to TTd and to α-syn

The effect of α-syn on the frequency of CD4+ T naive and memory cells was tested on PBMC obtained from a group of 8 HS (F/M = 4/4, age = 58.1 ± 14.5 years) and 6 PD patients (F/M = 2/4, age = 76.7 ± 7.0 years, UPDRS Part III = 20.5 ± 3.1, H&Y = 1.8 ± 0.8) all treated with l-DOPA without (n = 4) and with DA agents (n = 2, in both cases rasagiline, in one case also ropinirole), with LED = 551.7 ± 140.1 mg/day.

**Figure 6:**
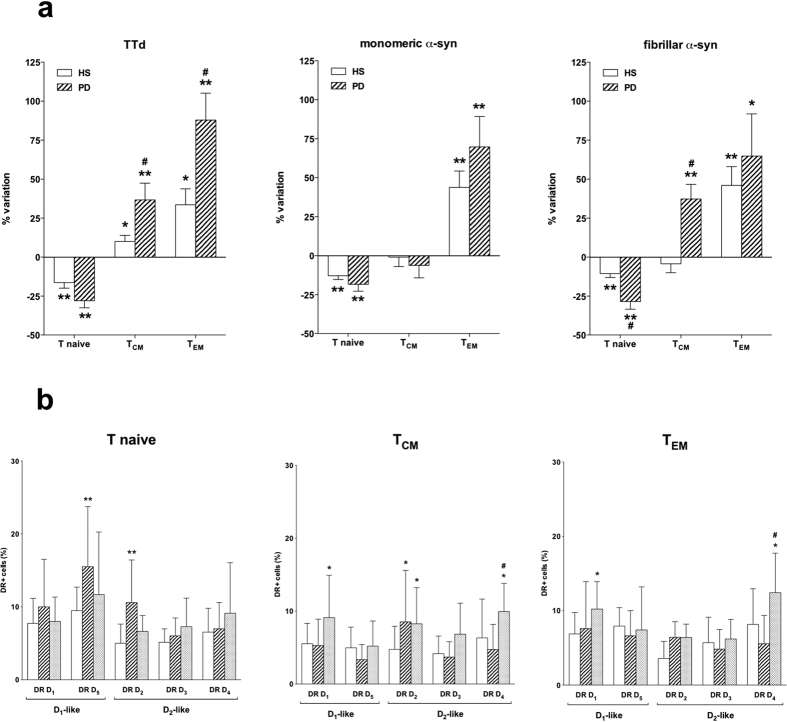
Effect of TTd, monomeric, and fibrillar α-syn on the frequency of CD4+ T naive and memory subsets. Panel (**a**) Effects of TTd (left), monomeric (middle), and fibrillar α-syn (right) in cells from HS (open columns) and PD patients (hatched columns). Data are expressed as percentage variation with respect to control conditions (without TTd or α-syn), and are means ± SEM of n = 6–8 separate experiments each performed in duplicate. * = P < 0.05 and ** = P < 0.01 vs. control conditions, and # = P < 0.01 vs. HS. Panel (**b**) monomeric (hatched columns), and fibrillar α-syn (shaded columns) on DR expression in T naive (left), T_CM_ (center) and T_EM_ cells (right) from 5 HS. Data are means ± SEM. * = P < 0.05 and ** = P < 0.01 vs control (open columns); # = P < 0.01 vs monomeric α-syn.

Incubation of PBMC for 48 h with TTd resulted in reduced CD4+ T naive and increased T_CM_ and T_EM_ in both HS and PD patients, however the increase in T_CM_ and T_EM_ was higher in PD patients (Fig. 6a, left). Incubation of PBMC for 48 h with either monomeric or fibrillar α-syn resulted in reduced CD4+ T naive cells and increased T_EM_ cells in both HS and PD, however in PD patients fibrillar α-syn also increased T_CM_ and induced a more pronounced reduction of T naive cells than in HS (Fig. 6a).

Both monomeric and fibrillar α-syn induced several changes in the expression of DR on CD4+ T naive and memory cells (Fig. 6b). In particular, monomeric α-syn increased DR D_5_ and D_2_ in T naive cells, and DR D_2_ in T_CM_, while fibrillar α-syn increased DR D_1_, D_2_ and D_4_ in T_CM_ and DR D_1_ and D_4_ in T_EM_.

Co-incubation of PBMC with either DA, the D_1_-like DR agonist SKF-38393, or the D_2_-like DR agonists 7-OH-DPAT and PD-168,077 did not affect the frequency of CD4+ T naive and memory cells (see Supplementary Table S3,). SKF-38393 (1 μM) or the D_2_-like DR agonist pramipexole (1 μM) did not modify the effects of monomeric and fibrillar α-syn in PBMC of either HS and of PD patients (data not shown).

## Discussion

The main result of our study is the evidence supporting an extensive association between DR expression in T lymphocytes and motor dysfunction, as assessed by the UPDRS Part III score, which is commonly used to measure disease severity in the clinical setting^35^. Specifically, in total CD4+ T cells as well as in CD4+ T naive cells the expression of D_1_-like DR D_1_ and D_5_ decrease with increasing UPDRS Part III score. On the contrary, D_2_-like DR show changes only at the mRNA level in total CD4+ T cells, do not exhibit major changes in CD4+ T naive cells, but show a clear trend to increased expression with increasing UPDRS Part III score in T_CM_ and in T_EM_. This is the first study showing a connection between PD severity and DR expression on CD4+ T cells, suggesting that dopaminergic pathways in peripheral immune cells are actively involved in PD. In addition, we provided preliminary evidence that α-syn might affect CD4+ T memory cells, possibly to a different extent in PD patients in comparison to HS.

Our results are in line with previous studies showing decreased CD4+ T lymphocytes in PD patients^9^,^10^, and in particular with Saunders *et al*.^12^, who recently reported that in PD patients increased effector/memory CD4+ T cells correlated with increased motor dysfunction. In our study PD patients had decreased absolute count of CD4+ T naive cells, increased percentage of T_EM_ cells, and T_CM_ not different from those in HS. Indeed, our flow cytometric strategy^28^ allowed to distinguish between T_CM_, which mediate reactive memory by homing to T cell areas of secondary lymphoid organs, and T_EM_, which afford protective memory, by migrating to inflamed peripheral tissues and displaying immediate effector function^36^,^37^. At apparent variance with the study by Saunders *et al*.^12^, who reported that in PD patients effector/memory CD4+ T cells increased with the UPDRS Part III score, we did not identify any clear correlation between T naive/memory cells and the UPDRS Part III score. Saunders *et al*.^12^ however identified T memory cells by using CD45RO expression, and found increased CD4+ T memory cells only in PD patients with UPDRS Part III score ≥31, while in our study we enrolled only 7 patients with a score above 20, the highest score being 24, and nonetheless we identified increased T_EM_ in PD patients, possibly also thanks to the specific flow cytometry staining strategy which included the expression of CD45RA and CCR7, and allowed to distinguish between T_EM_ and T_CM_^28^. It remains however to be established whether the enhanced peripheral T memory function occurring in PD patients is mainly T_EM_, in line with the possibility that peripheral immune activation in PD has at least in part a protective role.

Concerning the general peripheral immune profile, we also observed reduced CD4+ T naive cells with increasing age in HS but not in PD patients. Reduction of T naive cells in elderly subjects is well described and is believed to result from thymic involution in combination with ongoing differentiation of T naive cells into antigen-experienced memory/effector cells^34^. In PD patients, the absence of correlations between T naive cell count and age, together with the reduced number of T naive cells in comparison to HS, is indeed in agreement with the hypothesis that PD is associated with increased peripheral immune exposure to antigens. A contribution by dysregulated thymic T cells development cannot be discarded, however, also in view of the lack of studies on thymic function during PD.

Although many immune cell subsets are dysregulated in PD, the key role of CD4+ T cells in the pathogenesis of the disease is supported by their presence, together with CD8+ T cells, in the brain in both postmortem human PD specimens and in the MPTP mouse model of PD, and evidence obtained in the animal model indicate that CD4+ T cells are determinants of T cell-mediated dopaminergic cell death^11^. Moreover, a recent study in MPTP-treated mice showed that CD4+ T cells are necessary for MPTP-induced neurodegeneration and that D_2_-like DR D_3_ expressed on T cells favor their activation and acquisition of the Th1 inflammatory phenotype^29^.

Our results show that mRNA expression of several DR are dysregulated in CD4+ T cells from PD patients: in particular, in comparison to cells from HS, in cells from PD patients mRNA for the D_1_-like DR D_5_ and for the D_2_-like DR D_3_ and D_4_ are decreased, and mRNA for the D_2_-like DR D_3_ is increased (Fig. 2a,b). Flow cytometry analysis of DR expression on CD4+ T cell membranes provides however a more homogeneous picture (Fig. 2c–f), with D_1_-like DR D_5_ clearly reduced by 30–49% in cells from PD patients. Interestingly, reduction of D_1_-like DR was evident for both DR D_1_ and D_5_ in CD4+ T naive cells (Fig. 3), while no difference occurred in T_CM_ or T_EM_ cells (see Supplementary Fig. S1 and S2).

Little information is available on the physiopharmacology of D_1_-like DR-operated pathways in T cells. D_1_-like DR D_5_ likely mediate the inhibitory effects of dopamine on proliferation and cytotoxycity of human CD4+ and CD8+ T cells^38^, however they also play a role in the inhibition of human CD4+ CD25^high^ regulatory T cells, thus resulting in a “suppression of the suppressors”^23^. Interestingly, *in vitro* in human naive CD4+ T cells, dopamine via D_1_-like DR shifted T-cell differentiation towards Th2, in response to stimulation with anti-CD3 and anti-CD28 mAb^24^. Reduced D_1_-like DR on CD4+ T cells in PD patients might thus lead to several effects, such as increased CD4+ CD25^high^ regulatory T cell function and increased Th1/Th2 balance. Saunders *et al*.^12^ however, reported impaired function of CD4+ CD25^high^ regulatory T cells from PD patients, thus suggesting that reduced D_1_-like DR may have no direct effects on this specialized cell subset. Indeed, our preliminary unpublished data from another protocol, included in this same research program and aimed at investigating DR expression on CD4+ T helper subsets, likely suggest that PD patients have a Th1-biased peripheral immune profile. This observation is in agreement with the previously reported increased ratios of IFN-γ-producing to IL-4-producing T cells in PD patients^8^, as well as with the role of D_1_-like DR on human CD4+ T naive cells which, according to Nakano *et al*.^24^, shift T-cell differentiation towards Th2. It can thus be suggested that reduced D_1_-like DR on CD4+ T naive cells in PD patients impair their ability to differentiate towards Th2, promoting a Th1-biased proinflammatory profile.

D_1_-like DR on CD4+ T lymphocytes, which are generally reduced in PD, also display a close correlation with PD patients motor dysfunction, as assessed by the UPDRS Part III score. Indeed, DR D_5_ expression diminishes with increased UPDRS Part III score, both at the mRNA level as well as in terms of percentage of CD4+ T cells which express the specific receptors (Fig. 4), a behavior which is evident also in CD4+ T naive cells, for both DR D_1_ and D_5_, but not in T_CM_ of T_EM_ (Fig. 5). By contrast, CD4+ T_CM_ and T_EM_ cells generally display a linear trend towards increased D_2_-like DR (Fig. 5 and Supplementary Fig. S6). Such close association with the UPDRS Part III score was not always parallel with the H&Y stage. In particular, in CD4+ T naive cells DR D_1_ and D_5_ decreased, and in T_CM_ and T_EM_ DR D_2_ and D_4_ increased with increasing UPDRS Part III score (Fig. 5 and Supplementary Fig. S6) but not with increasing H&Y scale stage (Supplementary Fig. S7–9). A likely explanation is that only one of the 7 subjects with UPDRS Part III > 20 is included among the 4 subjects with H&Y 2.5–3.0 (Table 2). Whether UPDRS Part III scale profiles the underlying immune dysfunction occurring in PD patients better than the H&Y scale remains to be established. Unfortunately, the only other study correlating CD4+ T cells and motor dysfunction in PD considered just the UPDRS Part III score^12^.

PD patients with more severe motor dysfunction (score > 20) have T_CM_ cells expressing 131–134% more DR D_2_ and 112–126% more DR D_4_ than cells from HS, and 64–100% more DR D_2_ 64–105% more DR D_4_ in comparison to cells from PD patients with score 1–10. The picture is similar with T_EM_, as PD patients with score > 20 have T_EM_ cells expressing 93–112% more DR D_2_ and 48–71% more DR D_4_ than cells from HS, and 71–111% more DR D_2_ and 22–38% more DR D_4_ in comparison to cells from PD patients with score 1–10. As a whole, it appears therefore that, with increasing motor dysfunction, D_1_-like DR decrease on CD4+ T lymphocytes and in particular on CD4+ T naive cells, while D_2_-like DR, increase specifically on CD4+ T_CM_ and also on T_EM_ cells.

As discussed above, reduced D_1_-like DR on CD4+ T naive cells may promote a Th1-biased proinflammatory profile, and the present results suggest that such trend increases with increasing motor dysfunction. There is on the contrary paucity of data regarding the role of D_2_-like DR on T lymphocytes, even if Levite *et al*.^39^ showed that activation of either DR D_2_ or D_3_ might induce T cell proliferation and adhesion. Of potential relevance for the present results, it was recently reported that, in the MPTP mouse model of PD, D_2_-like DR D_3_ expressed on CD4+ T cells are critical for T cell-dependent microglial activation^29^. If the same applied to PD patients and to D_2_-like DR-operated pathways as whole (as in PD patients DR D_2_ and D_4_, but not DR D_3_, correlated with motor impairment), increased D_2_-like DR in the more advanced stages of the disease might imply increased activation of the peripheral immune system, in turn triggering central neuroinflammation leading to neurodegeneration and disease progression. Nonetheless, such findings should be interpreted cautiously since studies also exist showing that at least stimulation of the D_2_-like DR D_4_ may result in quiescence of human T cells^40^. It is therefore necessary to clarify the role of individual DR in the modulation of memory T lymphocytes and in their relationship with microglia in PD. In addition, the eventual role of antiparkinson treatments on DR expression on T lymphocytes needs careful consideration, as discussed hereafter.

Comparison between PD-dn and PD-dt patients did not reveal any major differences in the peripheral immune profile. In particular, absolute numbers of CD3+ and CD4+ T cells were not different, although percentage CD3+ T cells were slightly higher in PD-dt patients while percentage CD4+ T cells were slightly higher in PD-dn patients (Table 4), and T naive, T_CM_ and T_EM_ were similar in the two patient populations (Fig. 1b,d). A remarkable difference was however found in D_1_-like DR D_1_ expression in CD4+ T cells and in particular in T naive cells (Figs 2,3), as PD-dt patients had higher expression of DR D_1_ in comparison to PD-dn. This difference might be of interest as PD-dn patients have on their T naive cells only 22–35% DR D_1_ in comparison to HS T naive cells, while PD-dt patients have on their T naive cells 41–63% DR D_1_ in comparison to HS T naive cells. Whether this is an effect of dopaminergic antiparkinson treatments cannot be established on the basis of the present results, also taking into account that no relationship was found between PD duration and/or LED and the immune profile or DR expession in CD4+ T cells and cell subsets. The hypothesis should be nonetheless taken into account, since - as above discussed - D_1_-like DR on human CD4+ T naive cells may shift T-cell differentiation towards Th2^24^. Provided that this role of D_1_-like DR on T naive cells has any clinical relevance, it might be predicted that in the study which we are presently performing, aimed at investigating DR expression on CD4+ T helper subsets in PD patients, we will find less Th1 cells in PD-dt patients in comparison to PD-dn patients.

α-Syn is the major component of Lewy bodies and a key factor in PD pathogenesis. Pathological α-syn released by degenerating neurons activates microglia to a proinflammatory profile^32^, and directs cell migration^41^. Efflux of α-syn from the brain to peripheral blood has been reported in mice and possibly in PD patients^42^, and it has been hypothesized that it might prime T cells that, in turn, would enter the brain and sustain microglia activation and neurodegeneration^43^. It has also been suggested that the presence of aberrant forms of α-syn in the periphery may represent a possible means for exposure as a neoantigen and subsequent activation of the adaptive immune system^44^.

In our study, we performed preliminary experiments aimed at assessing the effects of different forms of α-syn on CD4+ T naive and memory cells, in comparison to a well established recall antigen like TTd. We tested both monomeric and fibrillar α-syn since accelerated fibril formation by certain variants of α-syn are associated to PD pathogenesis^45^,^46^. As expected, TTd reduced the frequency of T naive cells while increasing T_CM_ and T_EM_. The effect on T_CM_ and T_EM_ was however more pronounced in cells from PD patients, possibly in line with the activated profile of peripheral immune system in PD. Interestingly, both monomeric and fibrillar α-syn induced a response which was qualitatively similar to the one evoked by TTd. Both PD patients and HS responded to the same extent to α-syn, with the only exception of fibrillar α-syn, which increased T_CM_ cells in PD patients but not in HS.

Available evidence of course does not allow to conclude that the responses to α-syn are actually due to recognition of the protein by T memory cells, although the response pattern is similar to that induced by TTd, and the ability of the peripheral immune system to recognize α-syn is also supported by the occurrence of specific antibodies in the serum of PD patients and HS^47^,^48^,^49^. Nonetheless, the ability of fibrillar α-syn to increase T_CM_ is suggestive, as these cells mediate reactive memory, by homing to T cell areas of secondary lymphoid organs and readily proliferating and differentiating to effector cells upon antigenic stimulation^36^,^37^.

It is also remarkable that incubation with α-syn affected DR expression on CD4+ T cells, and that in particular fibrillar α-syn induced increased expression of DR D_4_ in both T_CM_ and T_EM_, a finding which resembles increased D_2_-like DR in T_CM_ and T_EM_ of PD patients with more severe motor dysfunction. Whether increased expression of DR corresponds to increased responsiveness, and which consequences might be implied for the pro/antiinflammatory balance of peripheral (and possibly also central) immunity need to be carefully considered.

### Concluding remarks and perspectives

It is noteworthy that in the present study we were unable to find any association between dopaminergic substitution treatments and the peripheral immune profile. Possible explanations include that l-DOPA may undergo conversion to dopamine only in the brain, and that dopaminergic receptor agonists are usually D_2_-like DR selective (pramipexole, ropinirole). Rotigotine is the only dopaminergic agonist currently used in PD that has comparable affinity for D_2_-like DR and at least for the D_1_-like D_5_, however our study enrolled only four subjects on rotigotine (out of a total of 53). Our study included also a group of newly diagnosed PD patients who never received dopaminergic treatments. The main difference between newly diagnosed and antiparkinson-treated subjects consisted in an even lower expression of D_1_-like DR D_1_ on total CD4+ T cells as well as in T naive cells in newly diagnosed patients, however it remains to be established whether the increased D_1_-like DR D_1_ expression in antiparkinson-treated patients is actually due to antiparkinson drugs and/or to other factors. In order to clarify this issue, we have already started a longitudinal study on a larger sample of drug naïve PD patients, who will be tested before and after pharmacological treatment.

Anyway, from a general point of view it is possible to conclude that dopaminergic substitution treatments have only minor, if any, impact on the peripheral immune system of PD patients, which on the other side shows profound differences in comparison to that of HS. In particular, specific differences related to dopaminergic pathways in immune cells definitely support the notion of a chronic peripheral immune activation in PD patients, which may affect disease severity. Immunotherapy is being increasingly regarded as an attractive strategy even in PD^44^, and it is therefore a priority to unravel the peripheral immune dysregulation occurring in PD patients, to plan adequate immunotherapeutic interventions. In addition, since antiparkinson therapy still lies mainly (if not only) on dopaminergic substitution therapy, detailed understanding of the role of dopaminergic pathways in the immune system might possibly allow a more appropriate use of available drugs, simply by better exploitation of their immunomodulating potential^14^,^15^,^16^,^50^.

## Materials and Methods

### Subjects

Peripheral venous blood samples were collected from patients with idiopathic PD^51^, either drug naive (PD-dn, i.e. PD patients who never received l-DOPA, DA agonists and/or other antiparkinson drugs) or on antiparkinson drug treatment (PD-dt), and from age- and sex-matched healthy subjects (HS). PD was diagnosed according to the United Kingdom Parkinson’s Disease Society Brain Bank Criteria. Patients and controls with a history of autoimmune or inflammatory disorders and those receiving chronic immunosuppressive treatment were excluded.

Participants were recruited through the Centre for Parkinson’s Disease and Movement Disorders of the Neurological Service at the Ospedale di Circolo of Varese, the Interdepartmental Research Center for Parkinson’s Disease of the Neurological Institute “C. Mondino” of Pavia, and the Movement Disorders Center of the University of Piemonte Orientale, Divisione di Neurologia, Ospedale Maggiore of Novara, Italy. Healthy subjects were spouses and caregivers of enrolled PD patients. The Ethics Committees of Ospedale di Circolo of Varese and Neurological Institute “C. Mondino” of Pavia approved the protocol and all the participants signed a written informed consent before enrollment. The study was performed according to the Declaration of Helsinki and to the relevant ethical guidelines for research on humans.

After enrollment, subjects were submitted to a complete examination. PD patients were staged according to the criteria of Hoehn and Yahr (H&Y)^52^ and evaluated by means of the Unified Parkinson’s Disease Rating Scale (UPDRS) Part III^53^. UPDRS Part II score was also assessed whenever possible. Data on patients and healthy controls were collected using standard data forms, which included demographics, diagnostic features, family history, primary diagnosis, PD features, UPDRS Part III score, and Hoehn and Yahr (H&Y) stage. Antiparkinson drug doses were recorded at the time of enrollment and l-DOPA equivalent doses (LED) were calculated according to established guidelines^54^.

Withdrawal of 30 ml venous blood was performed after a fasting night, between 8:00 a.m. and 10:00 a.m., in EDTA-coated tubes (BD Vacutainer). Tubes were subsequently coded and stored at room temperature until processing, which occurred within 24 hours after collection. Complete blood cell count with differential analysis was conducted on separate blood samples collected in EDTA-coated tubes (BD Vacutainer). Serum levels of dopamine were assayed by high-performance liquid chromatography with multielectrode electrochemical detection (HPLC-ED) according to a previously described method^23^.

### Reagents

Bovine serum albumin (BSA) and 4-(2-hydroxyethyl)-1-piperazineethanesulfonic acid (HEPES) were purchased from Sigma, Italy. RPMI 1640, heat-inactivated fetal bovine serum (FBS), glutamine, and penicillin/streptomycin were obtained from Euroclone, Italy. Ficoll-Paque Plus was from Pharmacia Biotech (Uppsala, Sweden). Purified mouse ab anti-human CD3 (code 555330, clone UCHT1, Mouse IgG1, κ) and purified mouse ab anti-human CD28 (code 555726, clone CD28.2, Mouse IgG1, κ) were obtained from Becton Dickinson, Italy. (±)SKF-38,393 hydrochloride (cod. D047), R(+)7-OH-DPAT hydrobromide (code H168), PD-168,077 maleate (code P233), pramipexol dihydrochloride (code A1237), and dopamine hydrochloride (code H8502) were all from Sigma, Italy. Human recombinant α-synuclein and its fibrillar form were a kind gift from Dr. Lars Kjær and Dr. Daniel Otzen (iNANO - Interdisciplinary Nanoscience Center, Aarhus University, Aarhus, Denmark), and were prepared as published before^55^.

### Flow cytometric analysis of naive and memory subsets of CD4+ T cells and of DR expression in whole blood

Analysis of CD4+ T naive and memory subsets and of DR expression was performed according to previously established method^28^. Briefly, 100 μl aliquots of whole blood were prepared and erythrocytes were removed by means of a lysis buffer ((g/L) NH4Cl 8.248, KHCO3 1.0, EDTA 0.0368). Samples were then centrifuged, supernatants were removed and cells were washed in PBS (pH 7.4) supplemented with 1% BSA (PBS/BSA) and resuspended in PBS/BSA. Total leukocytes were counted by means of a hemocytometer and cell viability, determined by the Trypan blue exclusion test, was always > 99%.

From each subject 7 aliquots of 100 μL were prepared: 5 were used for DR staining, 1 was used as control for the secondary PE-goat anti-rabbit (PEGAR) ab, and 1 was used as negative control (no ab). The staining protocol consisted of two steps. During the first step each aliquot was stained for one of the five DR by an indirect labeling procedure (primary ab + secondary ab labeled with PE). During the second step all the aliquots were incubated with a cocktail of anti-human CD3, CD4, CD45RA and CCR7 ab for the identification of T lymphocytes, CD4+ T lymphocytes and the following CD4+ T lymphocyte subsets: naive (CD3+ CD4+ CD45RA+ CCR7+ ), central memory (T_CM_, CD3+ CD4+ CD45RA-CCR7+ ), and effector memory (T_EM_, CD3+ CD4+ CD45RA-CCR7-). The complete list of ab used in the study is shown in Supplementary Table S1.

Acquisition was then performed on a BD FACSCanto II flow cytometer (Becton Dickinson, Milan, Italy) with BD FACSDiva software (version 6.1.3). Lymphocytes were identified by means of their classical forward scatter (FSC) and side scatter (SSC) signals and a minimum of 20,000 lymphocytes from each sample was collected in the gate. Data were analyzed with the FlowJo software (version 8.3.2). The results were finally expressed as absolute numbers (10^6^/ml) as well as percentage of positive cells (%).

### Isolation of peripheral blood mononuclear cells (PBMC)

PBMC were isolated from whole blood by using Ficoll-Paque Plus density gradient centrifugation. Cells were resuspended and, if necessary, any residual contaminating erythrocytes were lysed by addition of 5 mL of lysis buffer, followed by incubation for 5 min, during which samples were gently vortexed, and centrifugation at 100 g for 10 min at RT. Cells were washed twice in PBS by addition of 15 ml of PBS and centrifugation at 300 g and 10 min at RT, and resuspended at the final concentration of 10 × 10^6^ cells in 10 ml of RPMI/10% FBS for subsequent culture. Typical PBMC preparations contained at least 80% lymphocytes, as assessed by flow cytometry. Cell viability, assessed by the Trypan blue exclusion test was always > 99%.

### Real-time PCR assay of DR mRNA in CD4+ T cells

CD4+ T cells were isolated from PBMC by immunomagnetic sorting using Dynalbeads CD4 Positive Isolation kit (Life Technologies, code 11145D). Real-time PCR of DR mRNA was performed according to a previously reported method with modifications^56^. Briefly, to isolate RNA, at least 50000 CD4+ T cells were resuspended in *PerfectPure RNA lysis buffer* (5 Prime Gmbh, Hamburg, Germany), total RNA was extracted by *PerfectPure RNA Cell Kit*^*TM*^ (5 Prime Gmbh), and the amount of extracted RNA was estimated by spectrophotometry at λ = 260 nm. Total mRNA obtained from CD4+ T cells was reverse-transcribed using a random primer, high-capacity cDNA RT kit (Applied Biosystems). cDNA was then amplified with *SsoAdvanced™ Universal Probes Supermix* (BIORAD) for the analysis of DR D_2_, DR D_3_, and DR D_5_ gene expression, and with *SsoAdvanced™ Universal SYBR*^*®*^ *Green Supermix* (BIORAD) for analysis of DR D_1_, and DR D_4_ gene expression. cDNA was assayed on StepOne^®^ System (Applied Biosystems). Real-time PCR conditions are shown in Supplementary Table S2.

Linearity of real-time PCR assays were tested by constructing standard curves by use of serial 10-fold dilutions of a standard calibrator cDNA for each gene, and regression coefficients (r^2^) were always > 0.999; a melting curve was also performed to check for specificity of DR D_1_ (melting temperature = 83.5 °C) and DR D_4_ (melting temperature = 90 °C). Gene expression level in a given sample was represented as 2^−ΔCt^ where ΔCt = [Ct (sample) - Ct (housekeeping gene)]. Relative expression was determined by normalization to 18 S cDNA. Analysis of the data were performed by StepOne software™ 2.2.2- Applied Biosystems).

### Frequency of CD4+ T naive and memory subsets in cultured PBMC

Isolated PBMC were cultured in RPMI/10% FBS for 48 h at 37 °C in a moist atmosphere of 5% CO_2_, without or with anti-CD3/anti-CD28 ab (0.1 μg/ml). Tetanus toxoid (TTd, 3 μg/ml), monomeric or fibrillar α-syn (both 500 nM) were added at the beginning of cell culture. Cells were finally harvested and stained for flow cytometric analysis of naive and memory subsets of CD4+ T cells, as described in section regarding flow cytometric analysis of naive and memory subsets of CD4+ T cells.

### Statistical analysis

Distribution of the values was assessed by the D’Agostino & Pearson normality test. Statistical significance of the differences between HS and PD patients and between PD-dn and PD-dt patients was then analyzed by means of two-tailed Student’s *t* test or by the Mann-Whitney test, as appropriate, for continuous variables, and by the Fisher’s exact test for categorical variables. Correlations among continuous variables were assessed by Pearson or Spearman correlation analysis. Differences between HS and PD patients categorized for UPDRS Part III score or H&Y stage were analyzed by ordinary one-way ANOVA or by the Kruskal-Wallis test, with either Holm-Sidak’s or Dunn’s adjustments for multiple comparisons, and trend analysis in PD patients was performed by ANOVA post test for linear trend. Calculations were performed using commercial software (GraphPad Prism version 5.00 for Windows, GraphPad Software, San Diego California USA, www.graphpad.com).

## Additional Information

**How to cite this article**: Kustrimovic, N. *et al*. Dopaminergic Receptors on CD4+ T Naive and Memory Lymphocytes Correlate with Motor Impairment in Patients with Parkinson's Disease. *Sci. Rep.* **6**, 33738; doi: 10.1038/srep33738 (2016).

## Supplementary Material

Supplementary Information

## Figures and Tables

**Table 1:** Comparison between HS and PD patients.

	HS	PD	P
**n**	28	53	
**Gender** (F/M)	11/17	17/36	0.625
**Age** (years)	68.8 ± 8.1	69.7 ± 9.5	0.545^a^
**UPDRS Part II** (score)^b^		7.2 ± 3.5	
1–10 (n)		35	
11–20 (n)		6	
**UPDRS Part III** (score)^c^		14.0 ± 6.0	
1–10 (n)		18	
11–20 (n)		27	
>20 (n)		7	
**H&Y scale** (stage)		1.6 ± 0.6	
1.0 (n)		21	
1.5–2.0 (n)		25	
2.5–3.0 (n)		4	

Data are means ± SD unless otherwise indicated.

**Notes:** a = by Mann-Whitney *U* test; b = data missing for 1 PD-dn and 11 PD-dt; c = data missing for 1 PD-dn patient.

**Table 2:** Comparison between PD-dn and PD-dt.

	PD-dn	PD-dt	P
**n**	16	37	
**Gender** (F/M)	7/9	10/27	0.337
**Age** (years)	65.6 ± 10.8	71.4 ± 8.4	**0.041**^a^
**UPDRS Part II** (score)	6.9 ± 4.7^b^	7.5 ± 2.6^b^	0.606^d^
**UPDRS Part III** (score)	9.9 ± 5.9^d^	15.6 ± 5.3	**0.002**^a^
**H&Y scale** (stage)	1.2 ± 0.4	1.8 ± 0.5	**<0.001**^a^
**LED** (mg/day)		459.4 ± 247.4	
**Drugs**			
l**-DOPA** (n)		27^e^	
**DA agonists** (n)		27^f^	
pramipexole (n)		19	
ropinirole (n)		4	
rotigotine (n)		4	
**Rasagiline** (n)		19	

Data are means ± SD unless otherwise indicated.

**Notes:** a = by Student’s *t* test; b = data missing for 1 PD-dn and 11 PD-dt; c = data missing for 1 PD-dn patient; d = by Mann–Whitney *U* test; e = 8 taking l-DOPA alone, and 19 taking l-DOPA+ DA agents; f = 10 taking DA agonists alone (4) or with rasagiline (6), and 17 taking DA agonists+ l-DOPA, without (6) or with rasagiline (11).

**Table 3:** Complete blood count, comparison between HS and PD patients. Data are means ± SD unless otherwise indicated.

	units	range	HS	PD	P
**RBC**	10^12^/L	4.50–6.00	4.9 ± 0.4	4.7 ± 0.4	0.100^a^
**hemoglobin**	g/dL	13.0–17.5	14.4 ± 1.1	14.2 ± 1.2	0.472^a^
**hematocrit**	%	42.0–54.0	43.4 ± 3.6	42.6 ± 3.3	0.439^a^
**MCH**	pg	27.0–32.0	29.8 ± 1.8	30.1 ± 2.3	0.253^a^
**MCHC**	g/dL	32.0–36.0	33.7 ± 2.8	33.4 ± 1.7	0.780^a^
**Platelets**	10^9^/L	150–450	238.2 ± 50.1	242.8 ± 69.1	0.853^a^
**WBC**	10^9^/L	4.30–11.00	6.9 ± 1.6	6.6 ± 1.7	0.310^a^
***lymphocytes***	10^9^/L	1.50–5.50	2.12 ± 0.73	1.76 ± 0.48	**0.027**^b^
	%	10.0–45.0	30.8 ± 7.4	27.3 ± 6.8	**0.050**^a^
***monocytes***	10^9^/L	0.2–1.1	0.5 ± 0.1	0.5 ± 0.2	0.620^b^
	%	2.0–12.0	7.5 ± 1.9	7.5 ± 2.2	0.991^b^
***neutrophils***	10^9^/L	1.50–5.50	4.1 ± 1.1	4.1 ± 1.4	0.781^b^
	%	40.0–80.0	58.9 ± 8.0	62.4 ± 7.2	0.120^a^
***eosinophils***	10^9^/L	0.0–0.8	0.2 ± 0.3	0.2 ± 0.1	0.679^a^
	%	0.0–7.0	2.3 ± 1.6	2.3 ± 1.6	0.838^a^
***basophils***	10^9^/L	0.0–0.2	0.0 ± 0.0	0.0 ± 0.0	0.793^a^
	%	0.0–1.6	0.5 ± 0.2	0.5 ± 0.4	0.469^a^
***lymphocyte subsets***					
**CD3+**	10^6^/L		1515.0 ± 651.7	1241.0 ± 366.2	0.109^a^
	% of total lymph		70.8 ± 9.4	69.6 ± 8.7	0.549^a^
**CD4+**	10^6^/L		1012.0 ± 439.1	797.4 ± 263.3	**0.035**^a^
	% of CD3+		67.3 ± 10.8	64.7 ± 10.8	0.419^a^
**CD8+**	10^6^/L		334.0 ± 162.8	274.9 ± 151.5	0.134^b^
	% of CD3+		23.4 ± 9.3	22.0 ± 9.7	0.562^b^
**CD4**+ **/CD8+**	ratio		3.8 ± 2.9	4.0 ± 3.4	0.979^a^

Notes: Abbreviations: RBC, red blood cells; MCH, mean corpuscular hemoglobin; MCHC, mean corpuscular hemoglobin concentration; WBC, white blood cells. a = by Mann–Whitney *U* test; b = by Student’s *t* test.

**Table 4:** Complete blood count, comparison between PD-dn and PD-dt. Data are means ± SD unless otherwise indicated.

	units	range	PD-dn	PD-dt	P
**RBC**	10^12^/L	4.50–6.00	4.8 ± 0.4	4.7 ± 0.4	0.250^a^
**hemoglobin**	g/dL	13.0–17.5	14.4 ± 0.9	14.1 ± 1.3	0.456^a^
**hematocrit**	%	42.0–54.0	43.3 ± 2.9	42.2 ± 3.4	0.324^a^
**MCH**	pg	27.0–32.0	29.9 ± 1.7	30.2 ± 2.5	0.317^a^
**MCHC**	g/dL	32.0–36.0	33.3 ± 1.6	33.5 ± 1.8	0.847^a^
**Platelets**	10^9^/L	150–450	239.3 ± 48.8	244.4 ± 76.8	0.859^a^
**WBC**	10^9^/L	4.30–11.00	6.2 ± 1.6	6.7 ± 1.7	0.400^a^
***lymphocytes***	10^9^/L	1.50–5.50	1.63 ± 0.41	1.81 ± 0.50	0.222^b^
	%	10.0–45.0	27.1 ± 6.6	27.5 ± 6.9	0.841^b^
***monocytes***	10^9^/L	0.2–1.1	0.5 ± 0.2	0.5 ± 0.1	0.750^a^
	%	2.0–12.0	8.0 ± 2.6	7.2 ± 2.0	0.221^b^
***neutrophils***	10^9^/L	1.50–5.50	3.9 ± 1.4	4.2 ± 1.4	0.315^a^
	%	40.0–80.0	61.6 ± 7.7	62.7 ± 7.1	0.532^a^
***eosinophils***	10^9^/L	0.0–0.8	0.2 ± 0.1	0.2 ± 0.1	0.668^a^
	%	0.0–7.0	2.6 ± 2.0	2.2 ± 1.5	0.581^a^
***basophils***	10^9^/L	0.0–0.2	0.0 ± 0.0	0.0 ± 0.0	0.378^a^
	%	0.0–1.6	0.7 ± 0.4	0.4 ± 0.3	**0.019**^a^
***lymphocyte subsets***					
**CD3+**	10^6^/L		1115.9 ± 302.0	1295.0 ± 381.8	0.143^a^
	% of total lymph		65.9 ± 9.5	71.3 ± 7.9	**0.037**^b^
**CD4+**	10^6^/L		757.3 ± 208.3	814.8 ± 284.7	0.672^a^
	% of CD3+		69.2 ± 12.2	62.8 ± 9.6	**0.046**^b^
**CD8+**	10^6^/L		220.7 ± 134.6	299.7 ± 154.1	0.071^a^
	% of CD3+		19.5 ± 9.4	23.1 ± 9.8	0.220^b^
**CD4**+ **/CD8+**	ratio		5.3 ± 5.3	3.4 ± 1.8	0.053^b^

Notes: Abbreviations: RBC, red blood cells; MCH, mean corpuscular hemoglobin; MCHC, mean corpuscular hemoglobin concentration; WBC, white blood cells. a = by Mann–Whitney *U* test; b = by Student’s *t* test.
