# Supplementary material for: Dopaminergic Receptors on CD4+ T Naive and Memory Lymphocytes Correlate with Motor Impairment in Patients with Parkinson’s Disease
Source: Sci Rep. 2016 Sep 22;6:33738. doi: 10.1038/srep33738 (PMC5031979; doi:10.1038/srep33738)
Supplement: Supplementary Information [file srep33738-s1.pdf]

DOPAMINERGIC RECEPTORS ON CD4+ T NAIVE AND MEMORY LYMPHOCYTES CORRELATE WITH MOTOR IMPAIRMENT IN PATIENTS WITH PARKINSON'S DISEASE

Natasa Kustrimovic[1], Emanuela Rasini[1], Massimiliano Legnaro[1], Raffaella Bombelli[1], Iva Aleksic[1], Fabio Blandini[2], Cristoforo Comi[3], Marco Mauri[4], Brigida Minafra[2], Giulio Riboldazzi[4], Vanesa Sanchez-Guajardo,[5] Franca Marino[1], Marco Cosentino[1]

[1]Center of Research in Medical Pharmacology, University of Insubria, Varese (I); [2]Center for Research in Neurodegenerative Diseases, "C. Mondino", National Neurological Institute, Pavia (I); [3]Movement Disorders Centre, Neurology Unit, Department of Translational Medicine, University of Piemonte Orientale, Novara (I); [4]Department of Biotechnology and Life Sciences, University of Insubria, Varese (I); [5]Neuroimmunology of Degenerative Diseases group and AUidias pilot-center NEURODIN, department of Biomedicine, HEALTH, Aarhus University, Aarhus (DK)

Address for correspondence:

Marco Cosentino, MD PhD

Center of Research in Medical Pharmacology

University of Insubria

Via Ottorino Rossi n. 9

21100 Varese, VA, Italy

Via Rossi 9, 21100 Varese, Italy

Phone: +39 0332 217410, Fax: +39 0332 217409

E-mail: marco.cosentino@uninsubria.it

Supplementary Table S1. List of antibodies used in flow cytometric assays.

Target	Supplier	Species	Type	Conjugation	Dilution	Reference
human DR D_1	Calbiochem-Inalco	rabbit	IgG, polyclonal	n/a	1:100	324390
human DR D_5	Calbiochem-Inalco	rabbit	IgG, polyclonal	n/a	1:100	324408
human DR D_2	LifeSpan-Space Import Exp	rabbit	IgG, polyclonal	n/a	1:100	LS-C22924
human DR D_3	Calbiochem-Inalco	rabbit	IgG, polyclonal	n/a	1:100	324402
human DR D_4	LifeSpan-Space Import Exp	rabbit	IgG, polyclonal	n/a	1:100	LS-C22938
anti-rabbit IgG	R&D System, Space Import Exp	goat	IgG, polyclonal	phycoerythrin	undiluted	F0110
human CD3	Biolegend–Campoverde	mouse	IgG2a κ, clone OKT3	PerCPCy5.5	1:1	317336
human CD4	Becton Dickinson	mouse	IgG1 κ, clone RPA-T4	APC-Cy7	1:1	557871
human CD8	Becton Dickinson	mouse	IgG1 κ, clone RPA-T8	PE-Cy7	1:1	557746
human CD45RA	Biolegend–Campoverde	mouse	IgG2b κ, clone HI100	FITC	1:1	304106
human CCR7 (CD197)	Becton Dickinson	rat	IgG2a κ, clone 3D12	Alexa Fluor 647	undiluted	557734

Supplementary Table S2. Real-Time PCR conditions.

Gene	UniGene ID	Interrogated sequence *RefSeq/GenBank mRNA*	Detected coding transcripts	Amplicon context sequence	Chromosome location	Amplicon length	Annealing temperature (°C)	Efficiency (%)
DRD1	Hs.2624	NC_000005.9 NG_011802.1 NT_023133.13	ENST00000329144 ENST00000393752	GACAGGAAACAGGCAGTGAGGATACGAA CAGAGAAGTCCCTTCCACCACCAGCCCAG TCCCGTCCATGGCAGAGGTGTTCAGAGTC CTCATCTTCCTAAGAGAAAGCACATCA	5:174870011-174870124	84	60	100
DRD2	Hs.73893	NC_000011.9 NG_008841.1 NT_033899.8	ENST00000346454 ENST00000362072 ENST00000544518 ENST00000542968 ENST00000538967 ENST00000355319	AGCAGGGTGACAATGAAGGGCACGTAGA AGGAGACGATGGAGGAGTAGACCACGAA GGCCGGGTTGGCAATGATGCACTCGTTCT GGTCTGCGTTATTGAGTCCGAAGAGGAG	11:113286246-113287609	83	60	103

DRD3	Hs.121478	NC_000003.11<br>NG_008842.1<br>NT_005612.16	ENST00000460779<br>ENST00000467632<br>ENST00000295881<br>ENST00000383673<br>ENST00000281274	TTAAGGATGCTGGCTGTACACATCATGAC<br>ATCCAGGGTGACAAAAACATCACAGCAA<br>ATGCGGCTGAAATTCCAGACTCCACCTGT<br>CACCTCCAGGTATACCACCCAGGGCATCA<br>CCAAGGTGGCCACCAGCAAGTCTGCCACA<br>GCCAGGCTCACTA CTAAGTAGT	3:113878626-113890646	136	60	100
DRD4	Hs.99922	NC_000011.9<br>NG_021241.1<br>NT_009237.18	ENST00000176183	GCTGTGCTGGACGCCCTTCTTCGTGGTGCA<br>CATCACGCAGGCGCTGTGTCCTGCCTGCT<br>CCGTGCCCCCGCGGCTGGTCAGCGCCGTC<br>ACCTGGCTGGGCTACGTCAACAGCGCCCT<br>CAACCCCGTCATCTACACTGTCTTCAACGC<br>CGAGTTCCGCAACGTCTTCCGCA	11:640411-640580	140	60	98
DRD5	Hs.380681	NC_000004.11<br>NG_012024.1<br>NT_006316.16	ENST00000304374	TTTTAAACAGCAGGTTGTGTGTGTGTGCA<br>GTGATGTGGTGGGAGCACAGCTTTCCTGG<br>GTCTGGATTCCCGTGGCTTTGTGCTTATGT<br>CATTTCTTCTCTCTGTGCTGGTGGGGGCCT<br>CTTTACCATAGCTTAAG	4:9785451-9785585	105	60	97

RPS18	Hs.627414	NC_000006.11<br>NT_007592.15<br>NT_113891.2<br>NT_167245.1<br>NT_167247.1<br>NT_167248.1<br>NT_167249.1	ENST00000454021<br>ENST00000486781<br>ENST00000484321<br>ENST00000211372<br>ENST00000477055<br>ENST00000476288<br>ENST00000439602<br>ENST00000474973<br>ENST00000457341<br>ENST00000494232<br>ENST00000434122	GTGGAACGTGTGATCACCATTATGCAGAA<br>TCCACGCCAGTACAAGATCCCAGACTGGT<br>TCTTGAACAGACAGAAGGATGTAAAGGAT<br>GGAAAATACA	6:33243742-<br>33243838	67	60	98

Supplemental Table S3. Effect of dopamine (a) and of dopaminergic agonists (b) on the frequency of CD4+ T naive and memory cells in cultured human PBMC. Data are means±SEM of 3 separate experiments. Frequency is expressed as percentage of total CD4+ T cells. P always >0.05 vs. respective controls.

a.

	**T naive**	**T_{CM}**	**T_{EM}**
**Control**	50.9±5.2	25.0±5.5	19.3±3.5
**Dopamine**			
1 pM	52.0±5.5	26.9±5.3	17.0±2.2
100 pM	51.8±4.5	25.1±4.8	18.3±1.5
10 nM	52.8±5.6	26.5±4.2	16.8±3.5
1 μM	52.7±5.1	27.9±7.3	15.3±2.2
100 μM	49.7±5.6	20.5±4.1	23.5±2.8

b.

	**T naive**	**T_{CM}**	**T_{EM}**
**Control**	40.6±6.3	28.3±4.3	26.7±1.8
**SKF-38,393**			
100 nM	40.8±5.8	30.4±5.6	24.3±0.8
1 μM	41.7±5.9	29.8±5.3	23.9±1.1
10 μM	40.5±5.8	27.7±4.8	27.2±2.1
**7-OH-DPAT**			
10 nM	39.9±5.9	30.9±5.6	24.5±0.8
100 nM	40.5±6.6	27.4±4.6	27.1±2.5
1 μM	41.5±6.2	32.3±6.9	21.9±4.3
**PD-168,077**			
1 nM	40.7±6.2	29.0±5.3	25.6±2.5
10 nM	40.6±6.1	31.4±5.6	23.7±1.6
100 nM	41.1±6.2	31.7±4.5	23.2±2.1

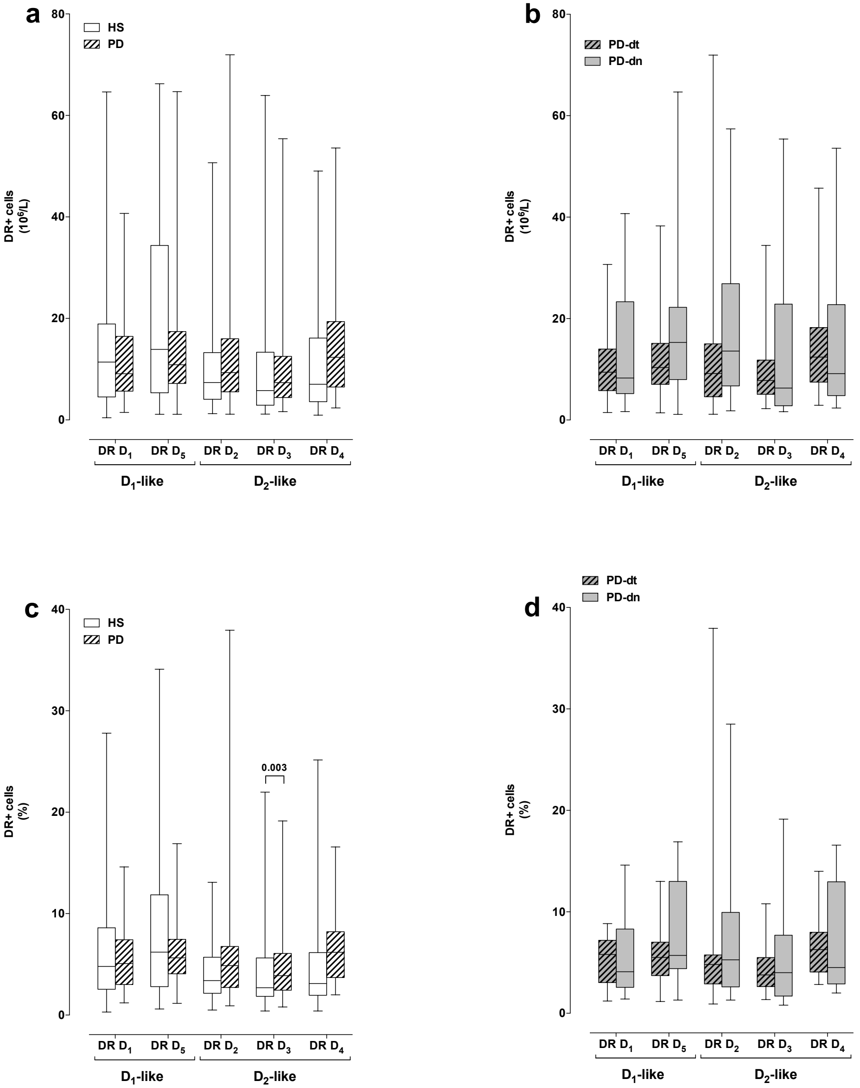

Supplementary Fig. S1 DR expression in CD4+ TCM cells from HS and from PD patients. DR+ cells are shown as absolute numbers (panels a and b) and as percentage of total CD4+ cells (c and d). Data are shown as medians with 25°-75° percentiles (boxes) and min-max values (whiskers). Comparisons are shown between HS and PD patients as a whole (a and c) and between drug naive (PD-dn) and drug treated (PD-dt) patients (B and D). Differences were analyzed by means of two-tailed Student's *t* test or by Mann-Whitney test, as appropriate. P values less than 0.05 are indicated in the graphs.

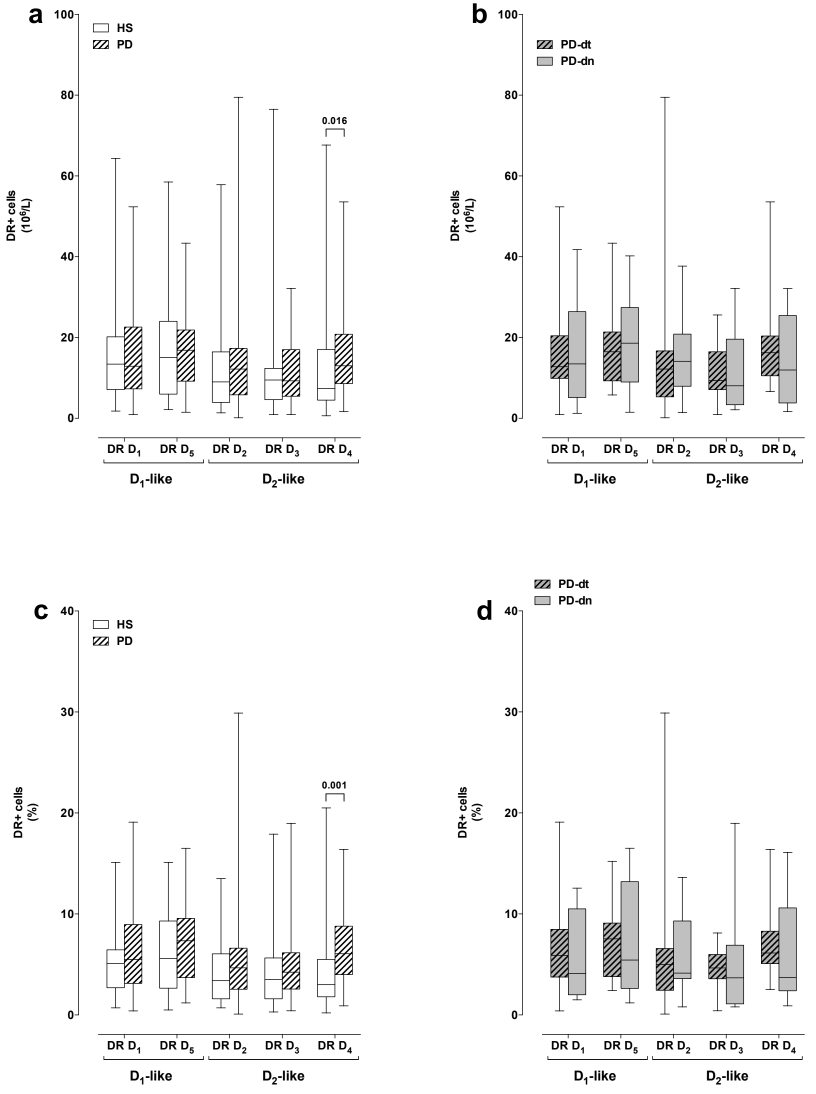

Supplementary Fig. S2 DR expression in CD4+ TEM cells from HS and from PD patients. DR+ cells are shown as absolute numbers (panels a and b) and as percentage of total CD4+ cells (c and d). Data are shown as medians with 25°-75° percentiles (boxes) and min-max values (whiskers). Comparisons are shown between HS and PD patients as a whole (a and c) and between drug naive (PD-dn) and drug treated (PD-dt) patients (b and d). Differences were analyzed by means of two-tailed Student's *t* test or by Mann-Whitney test, as appropriate.

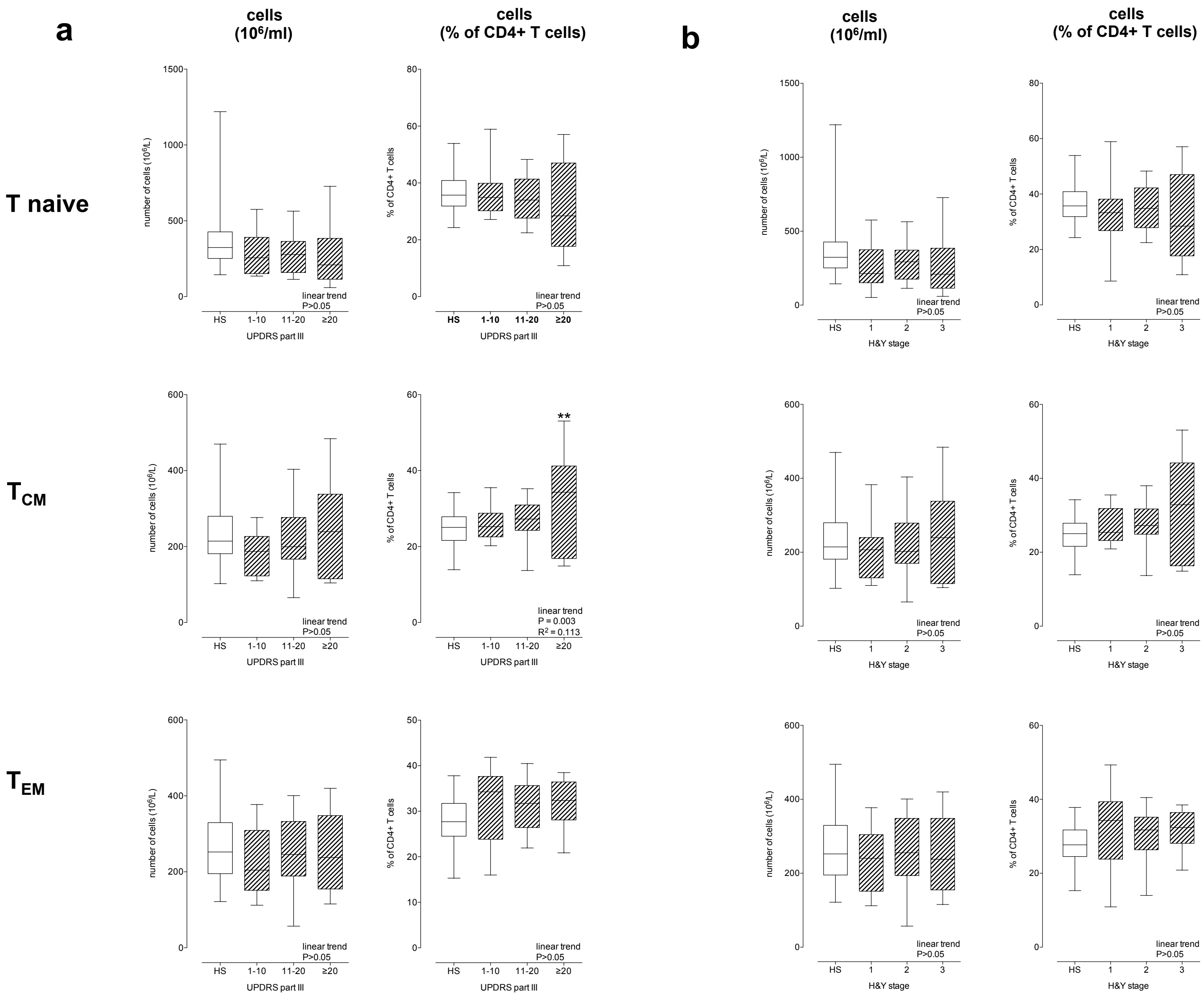

Supplementary Fig. S3 CD4+ T naive and memory cells and UPDRS-III score (a) and H&Y stage (b). Cells are shown as absolute numbers (left) and as percentage of total CD4+ T cells (right). Data are shown as medians with 25°-75° percentiles (boxes) and min-max values (whiskers). Differences between HS and PD patients were analyzed by parametric ANOVA or Kruskal-Wallis nonparametric ANOVA, with either Holm-Sidak's or Dunn's adjustments for multiple comparisons, where * = $P<0.05$. Trend analysis in PD patients was performed by ANOVA post test for linear trend.

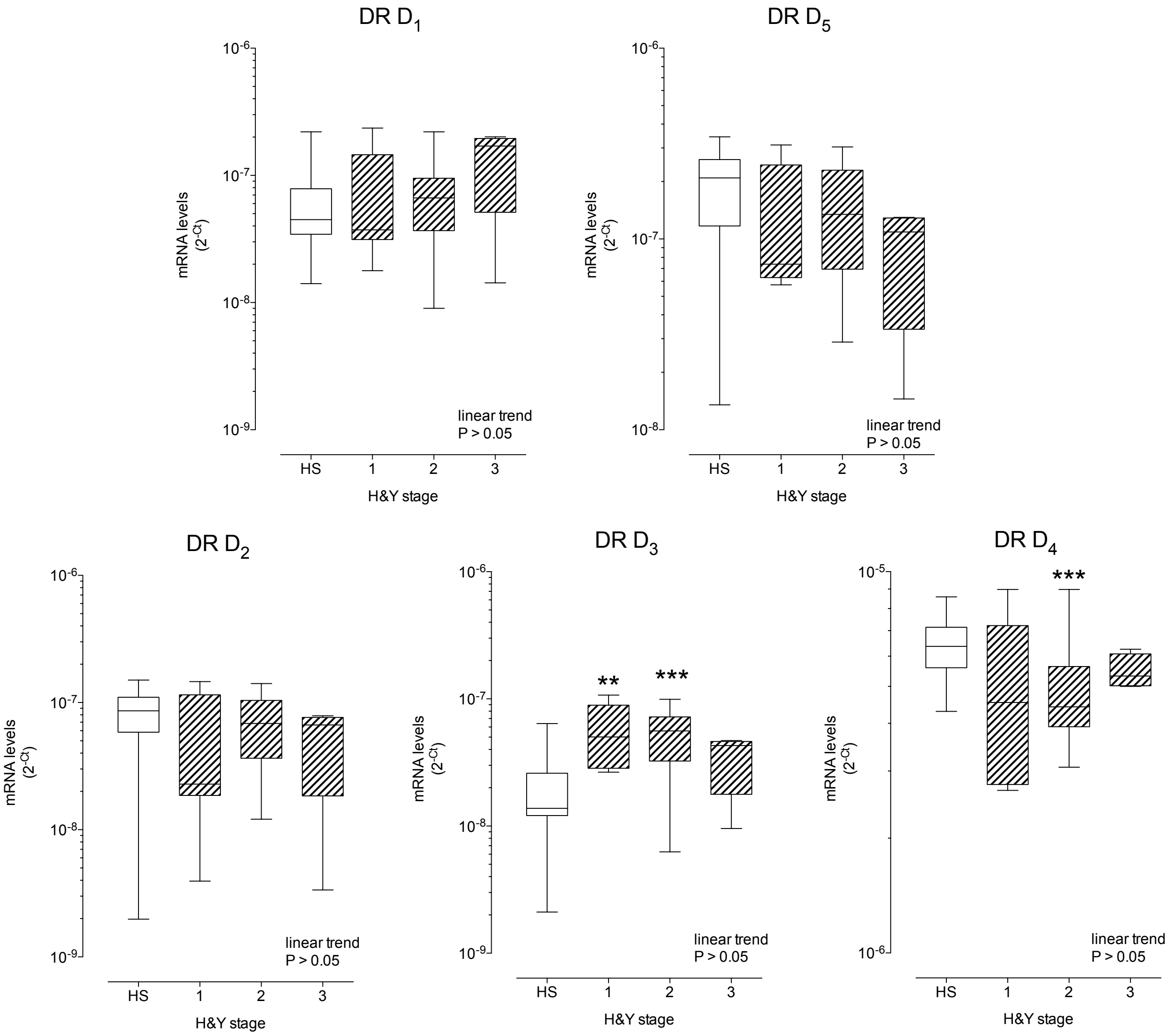

Supplementary Fig. S4 DR mRNA expression in CD4+ T cells and H&Y stage. Differences between DR mRNA levels in HS and in PD patients were analyzed by parametric ANOVA or Kruskal-Wallis nonparametric ANOVA, as appropriate, with either Holm-Sidak's or Dunn's adjustments for multiple comparisons, where * = P<0.05 and *** = P<0.001. Trend analysis in PD patients was performed by ANOVA post test for linear trend.

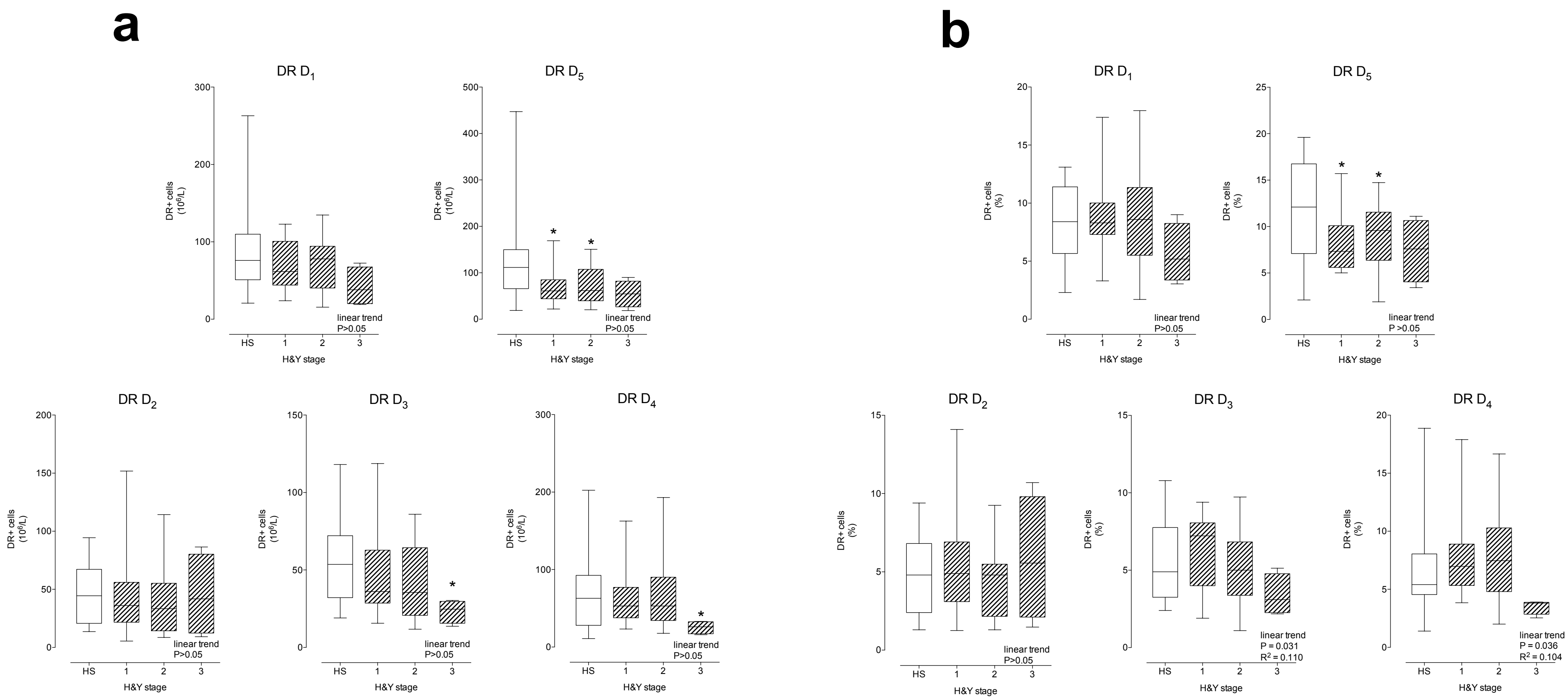

Supplementary Fig. S5 DR expression in CD4+ T cells and H&Y stage. DR+ cells are shown as absolute numbers (a) and as percentage of total CD4+ cells (b). Data are medians with 25°-75° percentiles (boxes) and min-max values (whiskers). Differences between DR levels in HS and in PD patients were analyzed by parametric ANOVA or Kruskal-Wallis nonparametric ANOVA, with either Holm-Sidak's or Dunn's adjustments for multiple comparisons, where * = $P<0.05$. Trend analysis in PD patients was performed by ANOVA post test for linear trend.

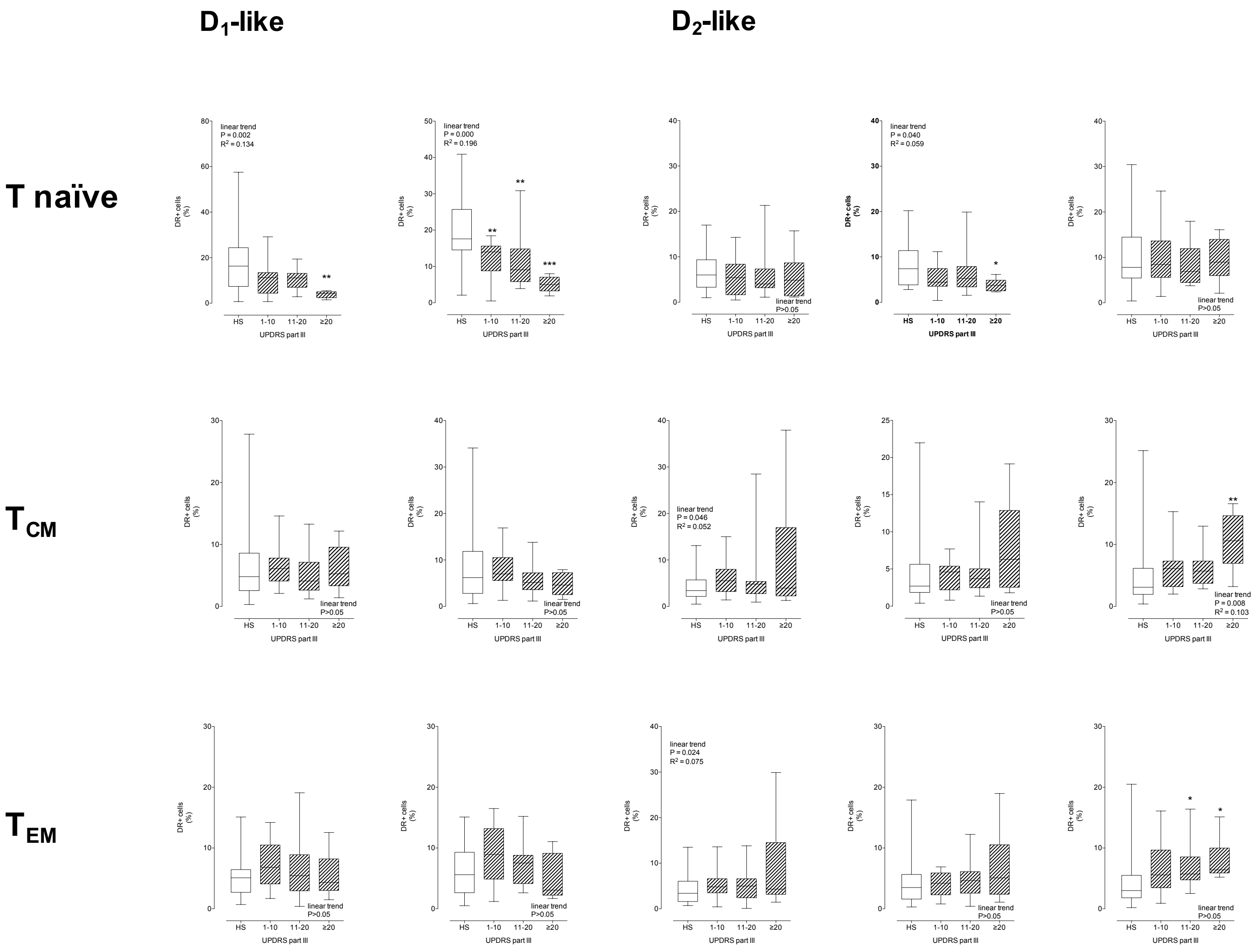

Supplementary Fig. S6 DR expression in CD4+ T naive (upper), T_{CM} (middle) and T_{EM} (lower) and UPDRS-III score. DR expression is shown as protein expression on the membranes of CD4+ T cells, expressed as percentage of CD4+ cells. Data are medians with 25°-75° percentiles (boxes) and min-max values (whiskers). Differences between DR levels in HS and in PD patients were analysed by parametric ANOVA or Kruskal-Wallis nonparametric ANOVA, with either Holm-Sidak's or Dunn's adjustments for multiple comparisons, where * = P<0.05, ** = P<0.01 and *** = P<0.001. Trend analysis in PD patients was performed by ANOVA post test for linear trend.

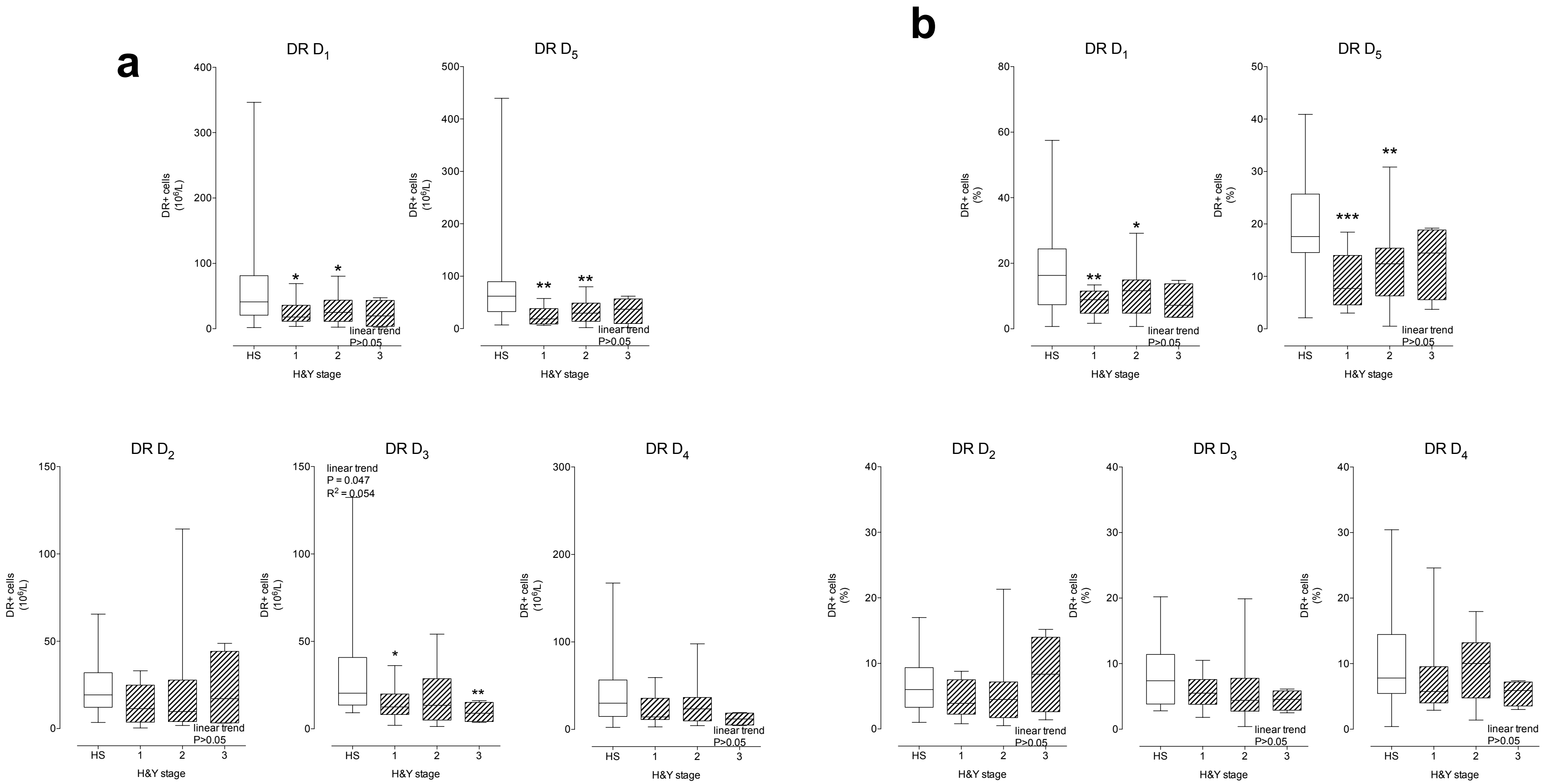

Supplementary Fig. S7 DR expression in CD4+ T naive cells and H&Y stage. DR+ cells are expressed as absolute numbers (A) and as percentage of total CD4+ T cells (B). Data are shown as medians with 25°-75° percentiles (boxes) and min-max values (whiskers). Differences between DR levels in HS and in PD patients were analyzed by parametric ANOVA or Kruskal-Wallis nonparametric ANOVA, with either Holm-Sidak's or Dunn's adjustments for multiple comparisons, where * = P<0.05, ** = P<0.01 and *** = P<0.001 . Trend analysis in PD patients was performed by ANOVA post test for linear trend.

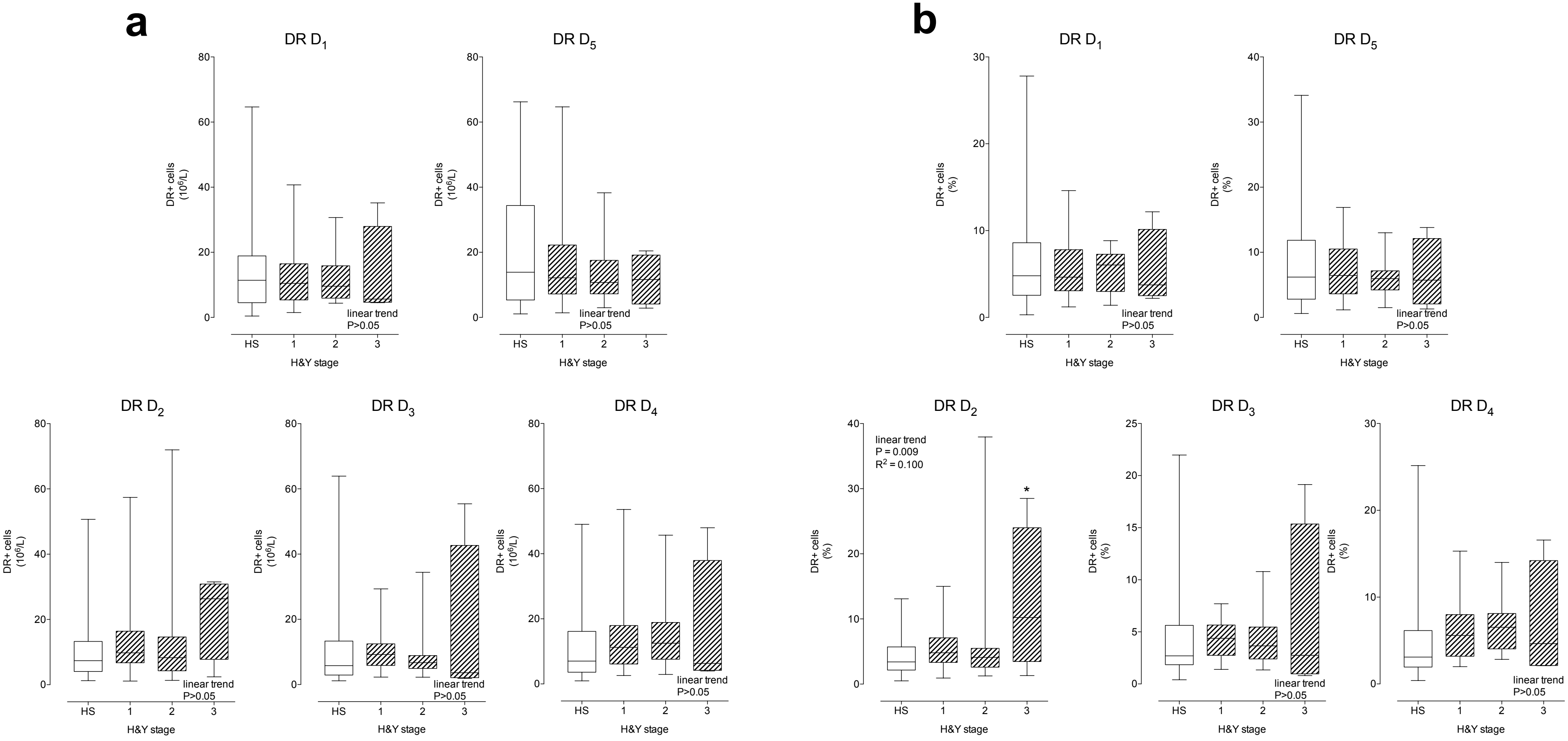

Supplementary Fig. S8 DR expression in CD4+ T_{CM} cells and H&Y stage. DR+ cells are expressed as absolute numbers (A) and as percentage of total CD4+ T cells (B). Data are shown as medians with 25°-75° percentiles (boxes) and min-max values (whiskers). Differences between DR levels in HS and in PD patients were analyzed by parametric ANOVA or Kruskal-Wallis nonparametric ANOVA, with either Holm-Sidak's or Dunn's adjustements for multiple comparisons, where * = P<0.05. Trend analysis in PD patients was performed by ANOVA post test for linear trend.

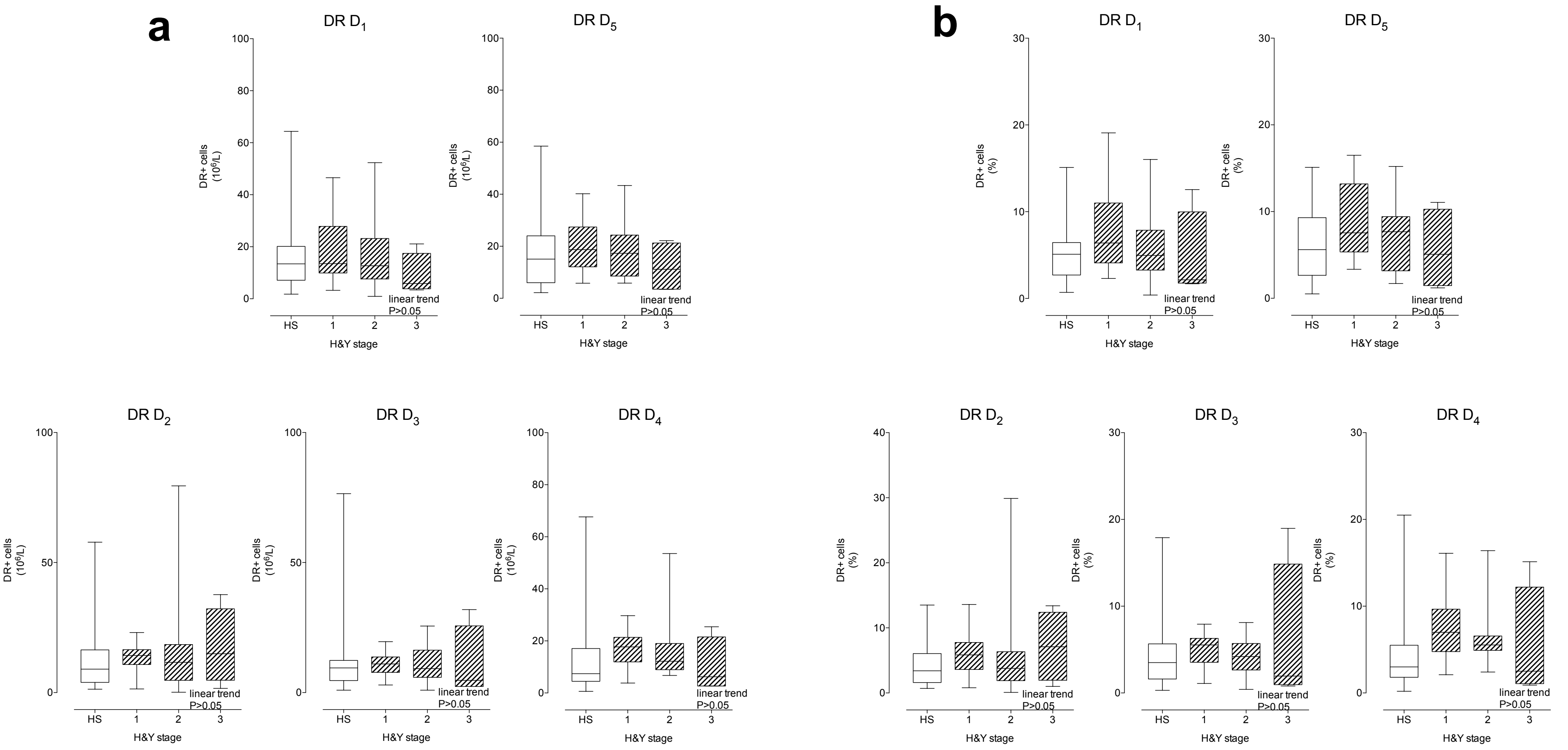

Supplementary Fig. S9 DR expression in CD4+ T_{EM} cells and H&Y stage. DR+ cells are expressed as absolute numbers (A) and as percentage of total CD4+ T cells (B). Data are shown as medians with 25°-75° percentiles (boxes) and min-max values (whiskers). Differences between DR levels in HC and in PD patients were analyzed by parametric ANOVA or Kruskal-Wallis nonparametric ANOVA, with either Holm-Sidak's or Dunn's adjustments for multiple comparisons. Trend analysis in PD patients was performed by ANOVA post test for linear trend.
